# Use of the revised World Health Organization cluster survey methodology to classify measles-rubella vaccination campaign coverage in 47 counties in Kenya, 2016

**DOI:** 10.1371/journal.pone.0199786

**Published:** 2018-07-02

**Authors:** Saleena Subaiya, Collins Tabu, James N’ganga, Abdulkadir Amin Awes, Kibet Sergon, Leonard Cosmas, Ashley Styczynski, Samson Thuo, Emmaculate Lebo, Reinhard Kaiser, Robert Perry, Peter Ademba, Katrina Kretsinger, Iheoma Onuekwusi, Howard Gary, Heather M. Scobie

**Affiliations:** 1 Global Immunization Division, Centers for Disease Control and Prevention, Atlanta, Georgia, United States of America; 2 Epidemic Intelligence Service, Centers for Disease Control and Prevention, Atlanta, Georgia, United States of America; 3 National Vaccines and Immunization Program, Ministry of Health, Nairobi, Kenya; 4 Kenya National Bureau of Statistics, Nairobi, Kenya; 5 World Health Organization Country Office, Nairobi, Kenya; 6 World Health Organization, Inter-country Support Team for Eastern and Southern Africa, Harare, Zimbabwe; 7 World Health Organization, Geneva, Switzerland; RIVM, NETHERLANDS

## Abstract

**Introduction:**

To achieve measles elimination, two doses of measles-containing vaccine (MCV) are provided through routine immunization services or vaccination campaigns. In May 2016, Kenya conducted a measles-rubella (MR) vaccination campaign targeting 19 million children aged 9 months–14 years, with a goal of achieving ≥95% coverage. We conducted a post-campaign cluster survey to estimate national coverage and classify coverage in Kenya’s 47 counties.

**Methods:**

The stratified multi-stage cluster survey included data from 20,011 children in 8,253 households sampled using the recently revised World Health Organization coverage survey methodology (2015). Point estimates and 95% confidence intervals (95% CI) of national campaign coverage were calculated, accounting for study design. County vaccination coverage was classified as ‘pass,’ ‘fail,’ or ‘intermediate,’ using one-sided hypothesis tests against a 95% threshold.

**Results:**

Estimated national MR campaign coverage was 95% (95% CI: 94%-96%). Coverage differed significantly (p < 0.05) by child’s school attendance, mother’s education, household wealth, and other factors. In classifying coverage, 20 counties passed (≥95%), two failed (<95%), and 25 were intermediate (unable to classify either way). Reported campaign awareness among caretakers was 92%. After the 2016 MR campaign, an estimated 93% (95% CI: 92%–94%) of children aged 9 months to 14 years had received ≥2 MCV doses; 6% (95% CI: 6%–7%) had 1 MCV dose; and 0.7% (95% CI: 0.6%–0.9%) remained unvaccinated.

**Conclusions:**

Kenya reached the MR campaign target of 95% vaccination coverage, representing a substantial achievement towards increasing population immunity. High campaign awareness reflected the comprehensive social mobilization strategy implemented in Kenya and supports the importance of including strong communications platforms in future vaccination campaigns. In counties with sub-optimal MR campaign coverage, further efforts are needed to increase MCV coverage to achieve the national goal of measles elimination by 2020.

## Introduction

During 2000–2016, measles elimination efforts prevented an estimated 20.4 million deaths; however, measles remains a leading cause of preventable deaths among young children globally [1]. In order to achieve the measles elimination goal adopted by all countries, ≥95% coverage with two measles-containing vaccine (MCV) doses is needed in each district [2].

In countries in the World Health Organization (WHO) African Region (AFR), only 24% of children were estimated to have received two MCV doses through routine health services in 2016 [3]. To reach missed children and achieve high population immunity against measles, most AFR countries conduct periodic MCV campaigns with a target of achieving ≥95% coverage in every district [2]. A combination measles and rubella (MR) vaccine is also being introduced in many AFR countries to control rubella and prevent congenital rubella syndrome (CRS), a spectrum of birth defects caused by rubella infection of pregnant women [4]. An estimated 39,000 CRS cases occurred in AFR in 2016 [5].

In Kenya, the WHO and United Nations Children’s Fund (UNICEF) estimates of national immunization coverage with the first and second MCV doses (MCV1 and MCV2) provided through routine immunization services at ages 9 and 18 months were 75% and 32% in 2016, far below the ≥95% target [6]. National MCV campaigns were conducted in 2002 for children aged 9 months to 14 years, and in 2006, 2009, and 2012 for children aged 9–59 months [7, 8]. A post-campaign cluster survey was conducted in 2002, which estimated national coverage as 94%, with two of seven provinces achieving <90% coverage [8, 9]. For the 2012 vaccination campaign, the estimated national coverage by survey was 90%, with provincial estimates ranging from 87%–95% [10].

As part of MR vaccine introduction in November 2016, the Kenya Ministry of Health (MOH) conducted a MR vaccination campaign in May 2016 targeting approximately 19 million children aged 9 months−14 years, or >40% of the total population, in order to control rubella, prevent CRS, and increase measles population immunity [11]. Since MCV campaigns began in Kenya, measles incidence has fallen from >100 cases per million annual population in the early 2000s to <10 cases per million annual population since 2013, with the last large outbreaks (thousands of cases) occurring between 2005–2007 and 2011–2012 [2, 7, 8, 12, 13]. Starting 6 months before the national MR campaign, a small measles outbreak of around 50 cases occurred in Mandera county (though obtaining reliable information from the outbreak area was challenging because of security issues), and outbreak response immunization targeting children 6 months–14 years of age was completed in Mandera and parts of neighboring Wajir county.

To evaluate vaccination coverage of the national MR campaign, a multi-stage cluster survey was conducted among children aged 9 months−14 years one month after the campaign started. This survey piloted methods recommended in the WHO 2015 draft manual for vaccination coverage cluster surveys [14]. This revised manual updates earlier guidance [15] with recommendations for conducting probability-based sampling, minimizing selection bias, and improving data quality [14]. In the revised manual, classification of subnational vaccination coverage was offered as an option for reducing sample size compared with an objective of estimating subnational coverage [14]. The primary objective of the post-campaign coverage survey in Kenya was to classify MR coverage in each of the 47 counties against the target of ≥95% coverage. Secondary objectives included estimating national coverage; assessing the effectiveness of communication strategies in promoting campaign awareness; identifying risk factors and reasons for non-vaccination; and estimating the proportions of children fully vaccinated (2 MCV doses), under-vaccinated (1 MCV dose), or unvaccinated against measles after the campaign.

## Methods

### Ethics statement

This evaluation was deemed a program evaluation activity by the Kenya MOH. The Office of the Associate Director for Science at the Center for Global Health, U.S. Centers for Disease Control and Prevention (CDC) determined the evaluation not to be human subjects’ research and approved it as a public health program evaluation activity, according to U.S. Department of Health and Human Services Human Subjects regulations and procedures. Because the evaluation was not human subjects’ research, a consent procedure was approved where verbal consent was obtained from participants at the beginning of the survey as part of a standard script, and refusal was documented on forms by survey teams (S1 and S2 Files).

### Study setting

Since 2013, the Kenyan government has devolved administrative functions from eight provinces to 47 counties. As of 2016, estimated total country populations ranged from 128,000 to 4,158,000 persons [16]. During 16–24 May 2016, a campaign was conducted with MR vaccine given to children aged 9 months–14 years at schools, health clinics, and other fixed sites. For the post-campaign survey, the MOH desired MR vaccination coverage results for each county because this was the relevant operational level for program implementation. A Demographic Health Survey (DHS) that was conducted in 2014 assessed routine immunization coverage at the county-level [17], which eliminated the need for inclusion in the post-campaign survey. The national MR vaccination coverage survey in Kenya was conducted during 16 June-7 July 2016, one month after the campaign start date, resulting in a possible range of 23–52 days between when children received the campaign vaccination and data was collected.

### Survey methodology

The sample size for county-level classification was selected using Annex B1 of the revised WHO Coverage Survey manual and designed so that a county with true campaign coverage ≥10% points below the 95% programmatic threshold for MR vaccination campaign coverage would have at least 90% probability of being classified as ‘failing’, i.e., a one-sided 95% upper confidence bound that fell below the 95% coverage threshold [14]. The lookup values in Table B-2 were: programmatic threshold = 95%; delta = 10%; alpha = 5%; and power = 90%. Based on these inputs, the required effective sample size was 77 children per county [14]. Adjusting for an estimated cluster survey design effect (DE) of 1.9 (based on an assumed intra-cluster correlation coefficient [ICC] of 0.10 and cluster size of 10 children), the target sample size was 147 children per county. With a binomial distribution, one can calculate the probability (Pr) of enrolling at least 147 eligible households in a county, given N total households sampled and visited, and a probability that a given household has at least one eligible child (p). We solved for N in each county by setting Pr = 90%, and a county-specific p = proportion of households in the 2014 DHS member listing that had an age eligible child (range: 0.43–0.85) [17]. N was then inflated for 10% non-response. Inclusion of these adjustments resulted in a required 225–510 household visits per county (15 clusters with 15–34 pre-selected households per cluster) to achieve the target sample size.Survey strata were the 47 counties. In the first stage, 720 clusters (15 per county, 30 in Nairobi) were selected using systematic random sampling with equal probability from the Kenya National Sample Survey and Evaluation Program (NASSEP V) frame. The NASSEP V frame contains clusters (enumeration areas) sampled probability proportion to size from the Kenya 2009 Population and Housing Census [16] and has household lists and maps for each cluster. The household lists and maps for 44% of selected clusters in 46 counties had been updated during 2014–2016, and the household lists and maps for 56% of selected clusters (including all clusters in Marsabit county) were created during 2012–2013 and had not been updated. In the second stage, a predetermined number of households that varied by county were selected using systematic random sampling with equal probability from the NASSEP V household lists for each selected cluster, resulting in inclusion of a total of 15,255 households. Households were defined as persons living together and eating from the same kitchen, and eligible households were defined as having had a child aged 9 months–14 years sleeping in the house the night before the survey.

Survey teams were comprised of trained staff from the Kenya National Bureau of Statistics (KNBS), and consisted of one supervisor and three interviewers that had relevant language abilities for their areas. Training consisted of one central 2-day training-of-trainers, and four regional 3-day trainings of supervisors and interviewers that included a field practical exercise. Each team was responsible for completing all the clusters in one county (with two teams assigned to Nairobi, for a total of 48 teams). Clusters found by teams to be inaccessible were not replaced. Survey teams used household numbers correlating to those found on cluster maps and household lists, along with the help of local guides to identify selected households. For vacant households, up to two revisits were made (total of three visits). In households with more than one caretaker with eligible children, all caretakers were listed, and one caretaker was selected using an electronically generated random number. From the selected caregivers, all eligible children aged 9 months–14 years at the time of the campaign were included in the survey.

Survey questionnaires were administered in Swahili or English using ODK Collect software on smartphones (Samsung Galaxy J2) and included questions on socio-demographic information, campaign information sources, vaccination status of children (MR campaign, routine MCV, previous MCV campaigns), and reasons for non-vaccination (S3 File). MR campaign vaccination status was documented by fingermark or caretaker’s recall, and routine MCV status was documented by vaccination card or caretaker’s recall. Teams were monitored daily by regional coordinators and central MOH and KNBS staff. Data were regularly uploaded during survey implementation and data monitoring was performed daily; in the event of a discrepancy, teams were contacted by short message service (SMS, or text message), email, or phone to rectify the entry.

### Data analysis

Data were analyzed using SAS v9.3 (SAS Institute, Cary, North Carolina). Sample weights were calculated to account for sampling probabilities at all stages of selection: master frame selection from the census, cluster selection, cluster segmentation, household selection, and the selection of one caretaker per household. The sample was post-stratified to account for differences in the urban and rural samples by county compared with the Kenya 2009 Population and Housing Census [16].

At the county level, vaccination coverage point estimates and one-sided upper and lower 95% confidence bounds were calculated using sample weights and a Taylor series linearization method to account for survey design. These one-sided 95% confidence bounds correspond to the upper and lower limits of a two-sided 90% modified Wilson confidence interval (CI). Coverage was classified using one-sided hypothesis tests against the program target of 95% coverage. The following classification rules were used: 1) if the lower confidence bound was greater than or equal to 95%, the county ‘passed’ (very likely ≥95%); 2) if the upper confidence bound was below 95%, the county ‘failed’ (very likely <95%); and 3) if the 95% threshold was contained within the upper and lower confidence bounds, the result was ‘intermediate’ (unable to confidently classify coverage as above or below 95% given the survey sample size). The proportion of the campaign target population residing in counties within the coverage classification categories was calculated.

Point estimates and Wilson 95% CIs for national vaccination coverage were calculated using sample weights and a Taylor series linearization method to account for survey design. Coverage by socio-demographic groups was calculated and compared using Rao-Scott chi-square tests. A proportion was estimated for the children previously unvaccinated and vaccinated for the first time. Proportions were also estimated for children fully vaccinated (≥2 MCV doses), under-vaccinated (1 MCV dose), or unvaccinated against measles after the MR vaccination campaign. Observed DEs, ICCs and effective sample sizes were calculated by county. Organ-pipe plots were constructed by plotting unweighted coverage by county and cluster [14]. To determine relative wealth quintiles, principle component analysis (PCA) was used to calculate a wealth index from indicators including household materials, source of lighting and cooking fuel, number of persons per room, and ownership of agricultural land and consumer goods, which were selected based on having a high degree of variability in the DHS (S1 Appendix) [17, 18]. Significant factors in our PCA included: type of roof (grass, wood), wall (cement/brick), flooring (cement, earth/sand), lighting source (electricity, kerosene), cooking fuel source (firewood, charcoal, gas), persons per room, and items owned (clock, radio, tv, sofa, cupboard). Descriptive analyses of sources of campaign information, location where child received vaccination, and reasons for non-vaccination were also conducted.

## Results

### Characteristics of the survey sample

Of the 15,255 sampled households, 15,147 (99%) households were visited; of these, 12,803 (85%) were non-vacant (5% temporarily absent, and 10% permanently vacant or demolished). Of the non-vacant households, 8,253 (64%) households had eligible children aged 9 months–14 years and consented to be interviewed, and 51 (0.4%) refused participation. Among eligible and consenting households, 388 (5%) households had more than one eligible caregiver requiring random selection of one caregiver, and 5,693 (69%) households had more than one eligible child, leading to the inclusion of 20,011 children. Overall, the surveyed households had a median size of 5 persons (range: 1–24), and 76% were in rural areas. About half of caregivers (51%) reported having at least some primary school education, and 27% reported secondary education or higher. Male children comprised 52% of our sample, and 76% of children reported attending school (Table 1).

**Table 1:** Socio-demographic characteristics of respondents in the measles-rubella vaccination coverage survey—Kenya, 2016.

**Households/ caregivers (n = 8,253)**			**No.**	**%**
Urban			1,941	24
Rural			6,312	76
Household head’s occupation				
	Unemployed, Retired, or Student		693	8
	Subsistence Farming		3,260	40
	Pastoralist		820	10
	Self- Employed		1,972	24
	Formal employment		1,346	16
	Other		102	1
Household head’s education				
	None		1,668	20
	Primary		3,780	46
	Secondary		2,698	33
	Other		107	1
Caregiver’s education				
	None		1,816	22
	Primary		4,225	51
	Secondary		2,126	26
	Other		86	1
Caregiver’s literacy				
	Illiterate		1,136	14
	Literate*		7,117	86
		Swahili & English	3,650	51
		Swahili	2,357	33
		English	65	1
		Other	1,045	15
Caregiver’s religion				
	Catholic		1,730	21
	Other Christian		5,010	61
	Muslim		1,217	15
	Other		296	4
			**Median**	**Range**
Caregiver’s age (years)**			35	(12–95)
Household size (persons)			5	(1–24)
Eligible no. of children per caregiver			2	(1–10)
**Children (n = 20,011)**			**No.**	%
Child’s sex				
	Female		9,694	48
	Male		10,317	52
Child’s age				
	9-59m		5,837	29
	5-9y		7,614	38
	10-14y		6,560	33
Child’s school attendance				
	Day school		14,992	75
	Boarding school		267	1
	No school		4,752	24

* Respondents were asked to select all languages for which they could read and write

** Age data were missing for 64 caregivers because of issue with electronic form

### Measles-rubella campaign coverage

Nationally, an estimated 95% (95% CI: 94%-96%) of children aged 9 months–14 years received the MR campaign dose, as assessed by fingermark or caregivers’ recall. Of the 20,011 children included, 8,665 (43%) were present at the time of the survey; of these, 1,703 (20%) children had visible fingermarks. MR vaccination coverage was significantly different (p < 0.05) by age group, school attendance, religion, caregivers’ education, caregivers’ literacy, household head occupation, household head education, and household wealth quintile; no difference was observed by urban/rural residence (Table 2).

**Table 2:** Estimated measles-rubella campaign vaccination coverage overall and by subpopulation—Kenya, 2016.

Variable			Coverage (95% CI)	No. Vaccinated	Total No.	P-value
Overall			95% (94%-96%)	19,001	20,011	-
	By fingermark		9% (8%-10%)	1,703		
	By caregiver’s recall		87% (85%-88%)	17,298		
Residence						
	Urban		95% (92%-97%)	3,856	4,074	p = 0.570
	Rural		96% (95%-96%)	15,145	15,937	
Child’s age						
	9–59 months		94% (93%-95%)	5,446	5,837	p<0.001*
	5–9 years		97% (96%-97%)	7,335	7,614	
	10–14 years		95% (94%-96%)	6,220	6,560	
Child’s sex						
	Female		96% (95%-97%)	9,234	9,694	p = 0.002*
	Male		95% (94%-96%)	9,767	10,317	
Child’s school attendance						
	Day school		97% (96%-98%)	14,569	14,992	p<0.001*
	Boarding school		90% (84%-94%)	235	267	
	Does not attend school		90% (88%-92%)	4,197	4,752	
Household head’s occupation						
	Unemployed, retired, student		93% (86%-96%)	1,570	1,678	p = 0.025*
	Subsistence farming		96% (95%-97%)	7,835	8,168	
	Pastoralist		90% (86%-93%)	2,315	2,592	
	Self-employed		96% (95%-97%)	4,229	4,390	
	Formal employment		96% (95%-97%)	2,814	2,929	
	Other		95% (87%-97%)	238	254	
Household head’s education						
	No education		92% (89%-94%)	4,326	4,750	p<0.001*
	Primary education		96% (95%-97%)	8,876	9,202	
	Secondary education		96% (94%-97%)	5,547	5,765	
	Other		94% (84%-98%)	252	294	
Caregiver’s education						
	No education		91% (88%-94%)	4,882	5,360	p<0.001*
	Primary education		96% (96%-97%)	9,924	10,293	
	Secondary education		96% (95%-97%)	3,988	4,122	
	Other		95% (87%-98%)	207	236	
Caregiver’s literacy						
	Literate		96% (96%-97%)	16,033	16,705	p<0.001
	Illiterate		89% (85%-93%)	2,968	3,306	
Caregiver’s religion						
	Catholic		96% (95%-97%)	3,855	4,037	p = 0.005*
	Other Christian		96% (96%-97%)	11,078	11,471	
	Muslim		90% (84%-94%)	3,351	3,683	
	Other		90% (85%-94%)	717	820	
Household wealth quintile						
	1 (lowest)		91% (88%-93%)	4,671	5,174	
	2		96% (94%-97%)	4,098	4,254	
	3		97% (96%-98%)	3,874	3,964	p<0.001
	4		97% (96%-98%)	3,445	3,545	
	5 (highest)		96% (94%-97%)	2,940	2,940	

* Statistically significant result by Rao-Scott chi-square test

**Fig 1:**
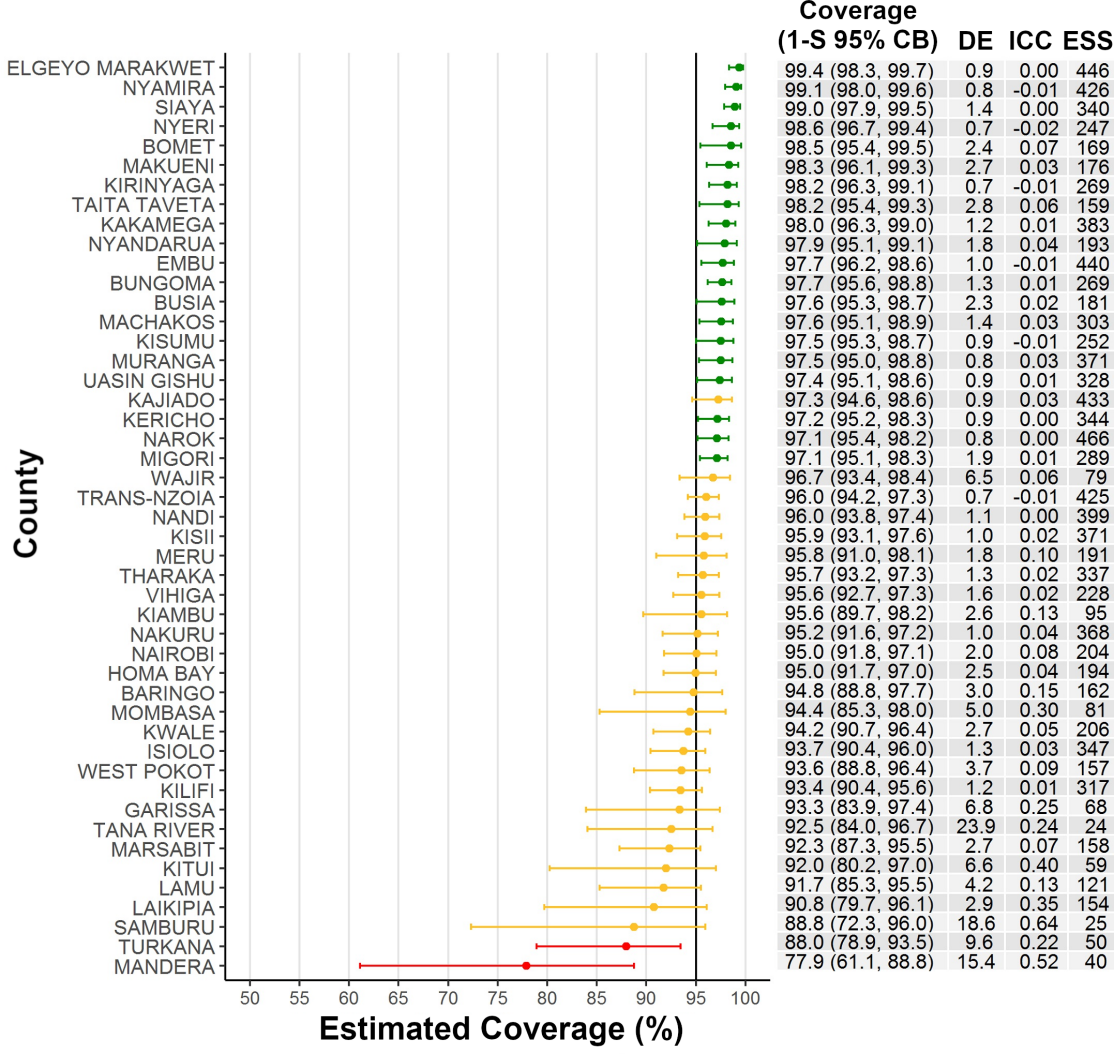
Classification of measles-rubella campaign vaccination coverage by county—Kenya, 2016. Coverage point estimates (one-sided upper and lower 95% confidence bounds [1-S 95% CB]) for children aged 9 months–14 years by country are graphed and printed in the columns to the right of the graph, along with the design effects (DE), intra-cluster correlation coefficients (ICC), and effective sample sizes (ESS = observed sample size / DE; where DE <1.0, a DE of 1.0 was used to calculate ESS). County coverages colored in green were classified as ‘passing,’ or likely to have coverage ≥95% (i.e., lower confidence bound was >95%). Coverages depicted in yellow were classified as ‘intermediate,’ or unable to confidently classify as above or below 95% given the survey sample size (i.e., upper and lower confidence bounds straddled the 95% threshold). Coverages shown in red were classified as ‘failing,’ or likely to have coverage <95% (i.e., upper confidence bound was below 95%).

**Fig 2:**
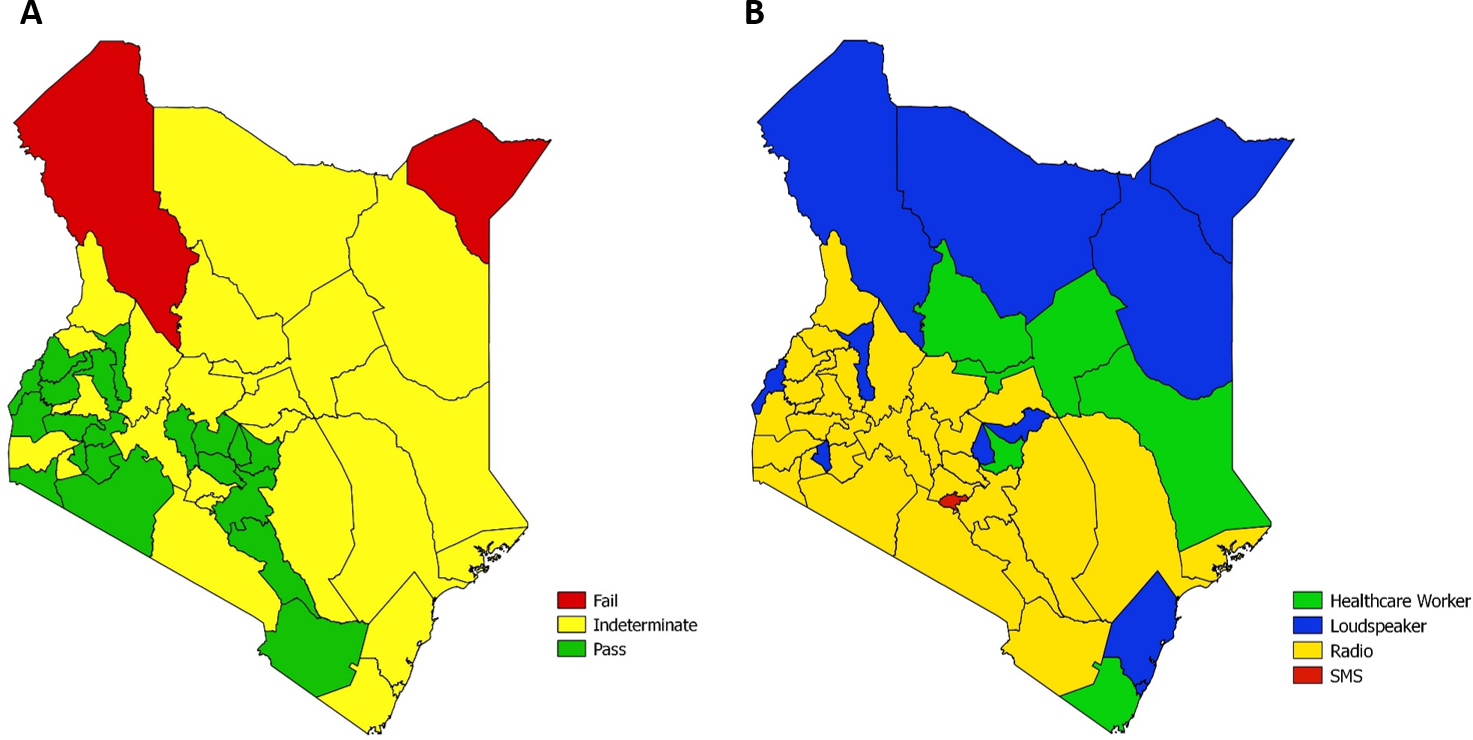
Map of measles-rubella (MR) campaign vaccination coverage and reported information sources by county—Kenya, 2016. (A) Using one-sided hypothesis testing against a programmatic threshold of 95%, MR campaign coverage of children aged 9 months–14 years was classified by county as either passing (likely to have coverage ≥95%), intermediate (unable to confidently classify as above or below 95% given the survey sample size), or failing (likely to have coverage <95%). Mandera is the northeast county depicted in red, which was the site of a measles outbreak in 2016, and Turkana is the other county depicted in red in the northwest. (B) The main source of MR campaign information that was most frequently reported by caregivers is depicted by respective county of residence.

Heterogeneity in the proportion of children who received the MR campaign vaccine dose by cluster varied substantially across countries (range: 0‒100%), with 71% of clusters having ≥95% of children vaccinated (S1 Fig). In classifying MR campaign coverage among Kenya’s 47 counties against a programmatic threshold of ≥95% coverage, 20 counties with 41% of the overall target population passed, 25 countries with 54% of the population were intermediate, and 2 counties (Mandera and Turkana) with 4% of the population failed (Fig 1 and Fig 2A). For counties that passed, coverage point estimates ranged 97%–99% with observed ICCs ranging -.02–0.06. Coverage estimates for intermediate counties ranged 89%–97%, with ICCs ranging -0.01–0.55. For the two counties that failed, coverage point estimates were 78% and 88%, with ICCs of 0.26 and 0.31, respectively (Fig 1).

### Campaign information

Awareness of the MR campaign among caretakers was 92%, with the most frequently reported sources of information being radio (32%), loudspeaker/PA (24%), and healthcare workers (15%) (Table 3). The most frequently reported primary source of campaign information varied by county. In general, loudspeaker and healthcare worker were most frequently reported as information sources in counties in the north and east, while radio was most frequently reported in the west and central regions; Nairobi was the only county where SMS reminders were the most frequently reported source of information (32%) (Fig 2B).

**Table 3:** Measles-rubella campaign awareness and main source of information reported by caregivers—Kenya, 2016.

Responses (n = 8,253)		No.	%
**Aware of campaign**		7,603	92.1
**Main source of information**			
	Radio	2,402	31.6
	Loudspeaker/PA	1,845	24.3
	Health worker	1,110	14.6
	Family/neighbor/friends	502	6.6
	TV	379	5
	Church/mosque/temple	326	4.3
	Community leader	311	4.1
	Mobile Phone/SMS	221	2.9
	Campaign vaccinator	139	1.8
	School	135	1.8
	House visitor	101	1.3
	Poster/banner	49	0.6
	Women/Youth group	44	0.6
	Newspaper	8	0.1
	Internet/Social Media	7	0.1
	Other	24	0.3

Caretakers reported their children received their MR campaign dose at school (59%), government health facilities (18%), home (12%), village meeting points (4%), temporary fixed sites (2%), churches/mosques (2%), markets (2%), and other sites (1%). The reasons most frequently reported by caregivers as to why their child was not vaccinated during the campaign were a lack of awareness about the campaign (26%), child’s absence during the campaign (22%), child’s illness (7%), visiting a post that was closed or had no vaccine (7%), forgetting to take their child (6%), and being too busy to take their child for the campaign dose (5%). It should be noted that for these two questions, an issue with a skip pattern in the electronic form resulted in missing data for children not present at the time of the interview: 11,853 (59% overall) missing for location of MR vaccination and 591 (55% overall) missing reason for non-vaccination during the campaign.

### Increase in measles vaccination coverage due to the campaign

Estimated MCV1 coverage through routine health services was 96% (95% CI: 94%-97%) among children aged 12–23 months, and estimated MCV2 coverage was 55% (95% CI: 51%-58%) among children 24–35 months, by vaccination card and caretakers’ recall; the availability of vaccination cards among the respective age groups was 26% for MCV1 and 13% for MCV2. Among children aged 12–23 months who missed MCV1, the reasons most frequently reported by caretakers were forgetting to take their child for vaccination (23%), lacking awareness about the need for vaccination (17%), and being too busy to take their child for routine vaccination (13%).

**Fig 3:**
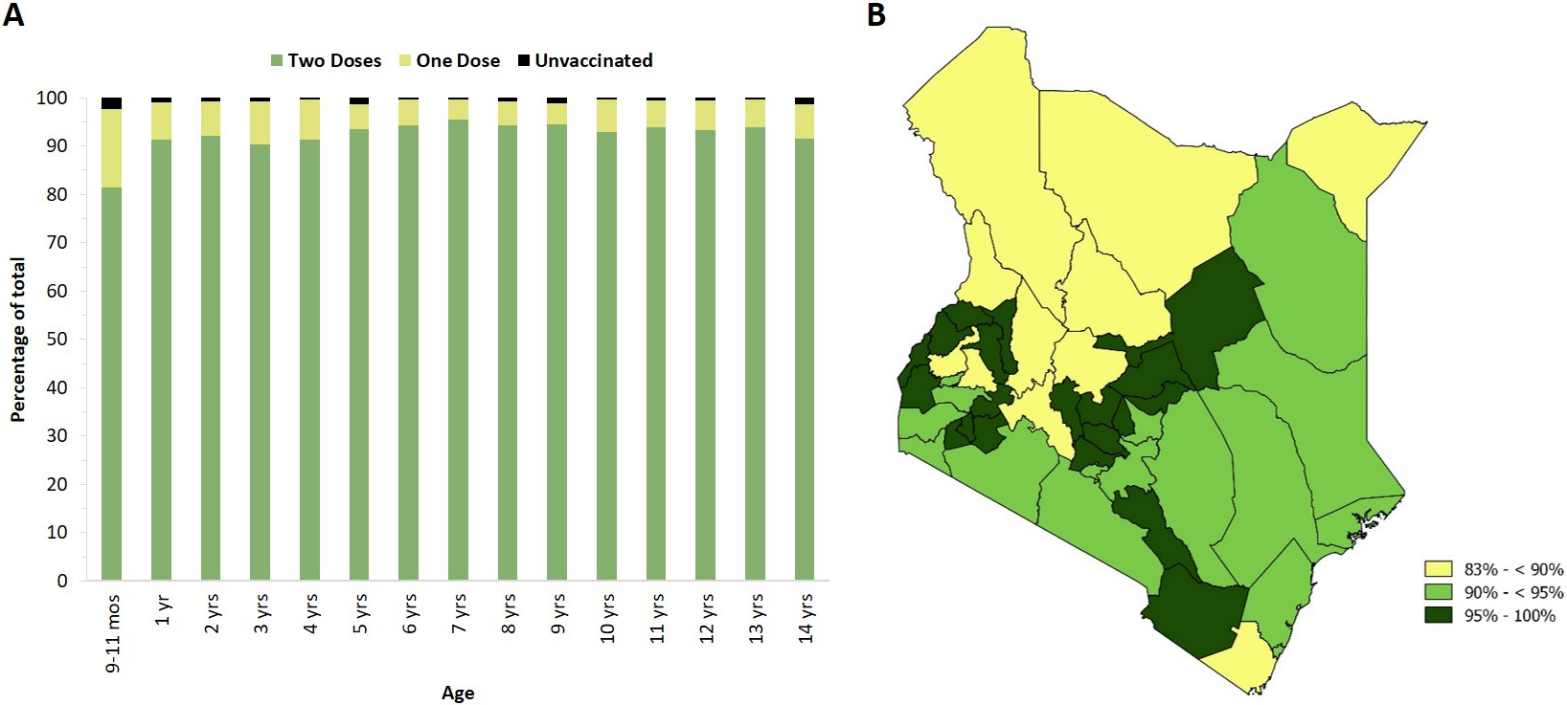
Estimated coverage with two doses of measles-containing vaccine (MCV) after the 2016 measles-rubella (MR) campaign in Kenya. (A) Graph of the number of MCV doses received by one-year age cohort, including the MCV1 and MCV2 doses provided by routine immunization services, the MR campaign dose, and previous measles campaign doses as documented by vaccination cards, campaign finger-markings and caregivers’ recall. (B) Map of two-dose MCV coverage of children aged 9 months–14 years by county.

Prior to the 2016 MR campaign, an estimated 5% (95% CI: 4%–6%) of children aged 9 months–14 years had never received a prior MCV dose, either through routine health services or a previous campaign. During the MR campaign in Kenya, an estimated 4% (95% CI: 4%–5%) of children aged 9 months–14 years received their first MCV dose. However, higher MR campaign coverage was observed among children aged 9 months–14 years who had received a prior MCV dose, compared to those who had no prior MCV dose (96% versus 85%, p <0.001). By the end of the 2016 MR campaign, an estimated 93% (95% CI: 92%–94%) of children aged 9 months to 14 years received ≥2 MCV doses; 6% (95% CI: 6%–7%) received 1 MCV dose; and 0.7% (95% CI: 0.6%–0.9%) remained unvaccinated (Table 4). Overall, two-dose MCV coverage ranged 90%–96% by one-year age cohort, with higher two-dose coverage among children aged ≥5 years (92%–96%) compared with children aged 1–4 years (90%–96%) and 9–11 months (82%) (Fig 3A). Two-dose MCV coverage by county ranged from 83%–100%, with many counties estimated to have <90% coverage clustered in the north and most counties with ≥95% coverage clustered in the central and western regions of the country (Fig 3B).

**Table 4:** Estimated proportion of children by age group and number of measles doses received at the end of the measles-rubella vaccination campaign—Kenya, 2016.

No. of doses*	Source	9–59 months		5–9 years		10–14 years		Total	
		No.	Percentage (95% CI)	No.	Percentage (95% CI)	No.	Percentage (95% CI)	No.	Percentage 95% CI
**0**	**Unvaccinated**	83	0.8 (0.6–1.2)	64	0.8 (0.5–1.2)	39	0.6 (0.4–0.9)	186	0.7 (0.6–0.9)
**1**	**MCV1**	243	3.9 (3.1–4.8)	93	1.1 (0.8–1.5)	122	1.6 (1.2–2.2)	458	2.1 (1.8–2.5)
	**MR campaign**	253	4.5 (3.7–5.5)	246	3.6 (2.9–4.6)	277	4.6 (3.8–5.5)	776	4.2 (3.6–4.9)
	**Prior campaign**	1	0.0 (0.0–0.1)	14	0.1 (0.0–0.3)	15	0.1 (0.1–0.3)	30	0.1 (0.1–0.2)
**2**	**MCV1 + MCV2**	51	1.1 (0.7–1.6)	0	-	0	-	51	0.3 (0.2–0.5)
	**MCV1 + MR campaign**	3,744	64.2 (62.2–66.0)	2,179	29.2 (27.2–31.4)	1,576	25.6 (23.7–27.6)	7,499	38.4 (36.9–40.0)
	**MCV1 + prior campaign**	12	0.2 (0.1–0.3)	108	1.4 (0.9–2.3)	164	2.8 (1.9–3.9)	284	1.5 (1.0–2.1)
	**MR + prior campaign**	12	0.2 (0.1–0.4)	156	2.0 (1.4–2.8)	156	2.5 (1.7–3.5)	324	1.6 (1.2–2.2)
**3**	**MCV1 + MCV2 + MR campaign**	869	15.5 (14.3–16.9)	0	-	0	-	869	4.6 (4.2–5.1)
	**MCV1 + MCV2 + prior campaign**	1	0.0 (0.0–0.1)	0	-	0	-	1	0.0 (0.0–0.0)
	**MCV1 + MR campaign + prior campaign**	537	8.9 (7.8–10.2)	4,754	61.7 (59.5–63.9)	4,211	62.3 (60.0–64.5)	9,502	46.2 (44.6–47.7)
**4**	**MCV1 + MCV2 + MR campaign + prior campaign**	31	0.7 (0.4–1.3)	0	-	0	-	31	0.2 (0.1–0.4)

* Includes the first and seconds measles-containing vaccine doses received through routine health services (MCV1, MCV2), the 2016 measles-rubella (MR) campaign dose, and prior campaign doses. Vaccination status was documented by card (for MCV1 and MCV2 only), finger marking (MR campaign only), or caregiver’s recall (for all doses). Where caregivers indicated a child received more than one prior campaign dose, only one prior campaign dose was included in the analysis to minimize the effect of recall bias. Inclusion of MCV2 doses was limited to children aged 18–35 months (after MCV2 introduction in Kenya).

## Discussion

The wide-age range MR campaign in Kenya reached the target of 95% national coverage, a major achievement and an improvement compared to the 2012 campaign (90% coverage by survey) [10]. The wide-age range campaign succeeded in rapidly increasing MR population immunity by vaccinating children against rubella for the first time and also by vaccinating a high proportion (85%) of children who had never before received a MCV dose. Lower campaign coverage was noted among children in certain vulnerable groups, including pastoralists (90%), lowest wealth quintile (91%), and associated with caregivers who were uneducated (91%) or illiterate (89%). Two counties, Turkana and Mandera, which represent 4% of the overall campaign target population, had campaign coverage that was classified as failing the ≥95% coverage threshold. This finding was troubling in light of the measles outbreak that occurred in Mandera several months before the 2016 MR campaign and may reflect underlying program weaknesses, as well as fatigue associated with conducting outbreak response immunization shortly before the national MR campaign. The coverage survey results were used as the basis for refining health systems strengthening activities, targeting interventions (e.g., immunization outreach and other equity interventions), improving data quality (i.e., in counties with divergent administrative coverage and survey results), as well as early planning for the next MR campaign.

Our choice of a three-level coverage classification scheme resulted in a majority of counties (53%) with MR campaign coverage classified as ‘intermediate’ [14]. Though the target number of eligible households (147) was surveyed in the vast majority of counties (91%), heterogeneity in campaign coverage and/or proximity to the programmatic threshold of 95% coverage resulted in 95% CIs that overlapped the target threshold of 95%. Classification of coverage was a lower cost alternative to estimation of coverage for counties, which are the relevant operational level for program implementation. However, ‘intermediate’ coverage classification had unclear implications for decision-making in terms of resource allocation for mop-up campaigns and future efforts to increase routine coverage [14].

For the campaign, reported administrative coverage, i.e., the percentage of children vaccinated among the target population, was 101%—6% higher than what was estimated by coverage survey (95%). Administrative coverage by county ranged from 26% lower to 36% higher than our survey estimates. Only one county had administrative coverage >10 percentage points lower than their respective survey estimate (Mombasa, 70% administrative coverage vs. 94% by survey), while 15 (32%) of 47 counties had administrative coverages >10 percentage points higher than survey estimates. Among the two failing counties, Turkana and Mandera, reported administrative coverage was 100%, as compared with survey estimates of 88% and 78%, respectively. While administrative coverage is used routinely to assess campaign coverage, it is known to be unreliable [19]. Our results underscore the importance of using rigorous probability based survey methodology to more reliably assess campaign vaccination coverage [20].

The high awareness of the MR campaign (92%) likely attributed to the campaign’s high coverage and related success in mobilizing parents to bring their children for vaccination. The Kenya MOH Health Promotion Unit worked closely with the National Vaccines and Immunization Program and partners to effectively implement various campaign communication strategies, including interpersonal communication (IPC) (e.g., by healthcare workers, home visitors and community/religious leaders) and mass media advertisements (e.g., TV, radio, loud speaker, newspapers, posters/banners, and social media). We noted regional differences in the main campaign information sources cited by caretakers (i.e., IPC versus mass media), which was likely related to differential access to mass media, as described in the 2014 DHS [17]. House-to-house mobilization was conducted in areas of three counties (Nairobi, Bungoma, Tharaka Nithi) by Red Cross volunteers during the 2016 campaign. This method was found to be effective for increasing awareness before and during the 2012 measles campaign in Kenya, in addition to using mass media advertisements [11, 21]. For the first time in Kenya, large number of SMS reminders about the MR campaign were sent to mobile phones in 22 (47%) of 47 counties, which might have helped to increase awareness, especially in Nairobi where SMS reminders were the most frequently reported source of campaign information. Collectively, these findings highlight the importance of employing a wide variety of well-planned communication methods to reach the target population.

In our survey, estimated routine MCV1 coverage among children born in Kenya during 2014–2015 (96%) was substantially higher compared with the 2014 DHS survey of those born during 2012–2013 (87%) [17]. The routine MCV2 coverage estimate of 55% among children vaccinated in 2015–2016 was higher than reported administrative coverage (28% in 2015 and 32% in 2016) [6]. The difference in MCV1 coverage between the two surveys may reflect the relatively lower vaccination card availability in our survey (25% in our survey vs. 75% in the 2014 DHS) and higher reliance on mother’s recall, which is known to be a less reliable source of vaccination history [17, 22]. In 2014 and 2015, the Kenyan government reported national stock-outs of vaccination cards through the WHO-UNICEF Joint Reporting Form which could potentially explain the low card availability observed in our survey; information from prior years relating to children included in the 2014 DHS was unavailable [23]. However, post-campaign survey monitors anecdotally observed a high proportion of available vaccination cards, with cards even available for older individuals (as old as 15–20 years). We suspect that the lower card availability observed in our survey may have been related at least in part to efforts by interviewers to save time by checking “caregiver’s recall” on the electronic form to skip subsequent questions requiring entry of vaccination dates, despite having reviewed information on cards.

An interim target of >80% MCV1 coverage in every district was adopted by AFR as a measles “pre-elimination” goal as well by the World Health Assembly as a global milestone to be achieved by 2015, while the global framework for measles elimination recommends a more stringent target of ≥95% two-dose MCV coverage in every district [24–26]. In Kenya, following the campaign, all counties achieved >80% coverage with two doses of MCV among children 9 months to 14 years, and 22 (47%) met the more advanced elimination target of ≥95% coverage with two MCV doses, by routine immunization or campaigns. The 2016 MR campaign represents substantial progress towards achieving measles elimination in Kenya by increasing two-dose coverage through routine immunization services or vaccination campaigns and by reducing the number of unvaccinated children. Similar to recent results of geographic modeling of MCV coverage in countries of the African Lakes Region, we noted persistent gaps in measles immunity in the northwestern region of Kenya and Mandera county [27]. To achieve measles elimination in Kenya, further effort is needed to increase MCV2 coverage through improving routine immunization services and to increase population immunity in underperforming counties.

In Kenya, the 2016 wide-age MR campaign was conducted in conjunction with the introduction of MR vaccine into routine immunization services at the end of 2016 in order to control rubella and prevent CRS [11]. Mathematical modeling has suggested that achieving ≥80% coverage through routine services and ≥60% coverage through initial catch-up campaigns of children aged ≤14 years will result in reduction of CRS cases—thresholds that all counties in Kenya exceeded [28]. With the introduction MR vaccine and continued progress towards measles elimination, Kenya will be on track to eliminate rubella and CRS [4].

Our study has limitations. While we used rigorous probability-based sampling methods and weighted analysis to ensure representative estimates for the population of Kenya, certain populations may have been underrepresented in our survey. The sampling frame was based on the 2009 census, so clusters with populations that disproportionately increased in size would be less likely to be included. In addition, five (0.7%) clusters were inaccessible due to insecurity or nomadic movement. Time and resource constraints prevented updating of cluster household lists prior to the survey, and households built after the cluster lists were last updated would have been excluded from selection (44% of cluster lists were updated during 2014–2016, and 56% during 2012–2013). We noted that 10% of selected households were either permanently vacant or demolished, compared with 7% in the 2014 DHS where household lists and maps for selected clusters were updated within the year preceding the survey [17]. Challenges related to reaching the school-age target of the MR campaign through a household survey resulted in a low proportion of children present at home during the survey. The low availability of campaign finger-markings (20%) was related to the poor quality of finger markers observed in the field during the 2016 campaign and time elapsed between the campaign and survey (roughly one month). To the extent caretakers’ recall was used as a source of routine or campaign vaccination history in our survey, misclassification of vaccination status may have occurred [22].

In future post-campaign cluster surveys, adequate time and budget should be allocated to allow for updating of cluster household lists to reduce potential sampling bias. To reduce the potential for misclassification of vaccination status, we recommend using a more durable marker of vaccination, like vaccination cards, and ideally recording the campaign vaccination separately from routine vaccination in a child’s home-based record [14, 29]. We also recommend greater attention to electronic questionnaire design and stressing the importance of documenting routine vaccine status by card during training and supervision of interviewers. Taking a photo of every routine vaccination card is recommended in the revised WHO coverage survey manual and would further facilitate documentation of vaccination dates [14]. For campaigns funded by Gavi, the Vaccine Alliance, only national level coverage estimates are required to validate the quality of campaign implementation [30]. If classification of subnational coverage is the desired survey objective for the country program, the need for the additional resources required to perform such a survey should be weighed against the potential program action that would result from the findings, which should be established in advance of the survey. Oversampling in high-risk areas or populations, either as part of a national or subnational survey, could potentially allow greater resolution in performance than that allowed for under a default scenario [30].

The 2016 wide-age range MR campaign in Kenya achieved high coverage (95%), which was a substantial achievement towards increasing measles and rubella population immunity. The successful MR campaign communication strategy employed in Kenya serves as a good example for future campaigns in the region; all countries should plan well in advance to implement diverse communication methods that motivate parents to bring their children to vaccination posts. Over the last decade, Kenya has demonstrated success in increasing overall measles population immunity through routine immunization services and campaigns [7–10]. However, vulnerable groups and county populations with persistently low coverage threaten the achievement of measles elimination. As part of MR vaccine introduction, we recommend focusing interventions to strengthen routine immunization services on counties with suboptimal MCV coverage revealed by this survey, including outreach strategies to underserved populations, towards the aims of achieving measles elimination by 2020 and preventing CRS.

## Supporting information

S1 File Program evaluation approval from Kenya Ministry of Health. (PDF)

S2 File Non-research determination and program evaluation approval from the U.S. Centers for Disease Control and Prevention. (PDF)

S3 File Questionnaire for the 2016 measles-rubella post-vaccination campaign survey in Kenya. (PDF)

S1 Fig Organ-pipe plots of unweighted measles-rubella campaign vaccination coverage by cluster and county—Kenya 2016. (PDF)
