# Supplementary material for: Use of the revised World Health Organization cluster survey methodology to classify measles-rubella vaccination campaign coverage in 47 counties in Kenya, 2016
Source: PLoS One. 2018 Jul 2;13(7):e0199786. doi: 10.1371/journal.pone.0199786 (PMC6028100; doi:10.1371/journal.pone.0199786)
Supplement: S1 File — (PDF) [file pone.0199786.s001.pdf]

# MINISTRY OF HEALTH
## OFFICE OF THE PRINCIPAL SECRETARY

**Telephone: Nairobi 254-020-2717077**
**Fax: 254-2719008**
**Email: pshealthke@gmail.com**

**AFYA HOUSE**
**CATHEDRAL ROAD**
**P. O Box 30016-00100**
**NAIROBI**

***When replying please quote:***

Ref: MOH/ADM/1/1/2

20th May 2016

The County Chief Officers of Health

Thro' Chairman
Council of Governors
Delta House – Westlands
**NAIROBI**

**POST MEASLES-RUBELLA SIAs COVERAGE SURVEY, JUNE 2016**

A global measles elimination goal is targeted for achievement in Kenya and other African countries by 2020. Due to concerted efforts, global measles-related deaths declined 75% during 2000-2013. However, measles virus still causes an estimated 150,000 deaths annually. In Kenya, over 1,500 cases were reported last year. Rubella virus infection in pregnant women can cause death of the unborn child and a spectrum of birth defects, called congenital rubella syndrome. Because of high rubella burden in Kenya, the Ministry of Health (MOH) plans to introduce a combination measles and rubella (MR) vaccine in 2016. This is preceded by an MR campaign targeting approximately **19 million children**, from **16th to 24th May 2016**. The goal of the campaign is to vaccinate >95% of children aged 9 months-14 years.

To evaluate and validate the vaccination campaign coverage, a multi-stage cluster survey is planned from **6th to 24th June 2016** to allow for estimation of coverage nationally. This will be implemented through the Kenya National Bureau of Statistics and will involve at visiting 15 NASSEP clusters.

The purpose of this letter is to inform you of this programmatic evaluation process and request for your support in provision of two vehicles to facilitate movement of the independent survey teams within your County.

The Drivers Allowance and Fuel for the vehicles will be provided through the survey teams. Kindly facilitate the process.

Thank you for your continued support.

Dr. Nicholas Muraguri
PRINCIPAL SECRETARY
