# Supplementary material for: Use of the revised World Health Organization cluster survey methodology to classify measles-rubella vaccination campaign coverage in 47 counties in Kenya, 2016
Source: PLoS One. 2018 Jul 2;13(7):e0199786. doi: 10.1371/journal.pone.0199786 (PMC6028100; doi:10.1371/journal.pone.0199786)
Supplement: S2 File — (PDF) [file pone.0199786.s002.pdf]

CGH HSR Tracking #: 2016-0101

Request for Project Determination & Approval – Center for Global Health (CGH)

This form should be used to submit proposals to the CGH Office of the Associate Director for Science/Laboratory Science (ADS/ADLS) for research/nonresearch determination and requirements for IRB review/approval.
Approval Chain: Investigator → Branch Chief/Country Director →Division ADS → CGH Human Subjects Mailbox

☑ **New Request** ☐ **Amendment** ☐ **Laboratory Submission**

Project Title: Cluster Survey Evaluation of a Measles-Rubella Campaign -- Kenya, June 2016	Project Location/Country(ies): Kenya
CDC Principal Investigator (SEV#: Saleena Subaiya, Heather Scobie	
CDC Project Officer(SEV#): Sue Chu	Division: GID · Telephone: 404-639-6084
Proposed Start Date (mm/dd/yyyy): 05/01/2106	Proposed End Date (mm/dd/yyyy): 12/31/2016

Collaborating Institutions (List other collaborating institutions in the protocol or in a separate document)

CoAg, Grant, or contract #:	GH0016535-01	IRB Exp. Date (if applicable): 01/27/2017
Title of CoAg, Grant, or Contract	National Stop Transmission of Polio (NSTOP) initiative supporting polio eradication in Africa,	
Supported Institution/Entity Name	NVIP, Kenya MOH	
Supported Institution/Entity FWA #	NA	FWA Exp. Date (mm/dd/yyyy):

Please check appropriate category and subcategory:

☑ **I. Activity is NOT human subjects research. Primary intent is public health practice or a disease control activity (Check one)**

- ☐ **A**. Epidemic or endemic disease control activity; if applicable, Epi-AID #
- ☐ **B**. Routine surveillance activity (e.g., disease, adverse events, injuries)
- ☑ **C**. Program evaluation activity
- ☐ **D**. Public health program activity*
- ☐ **E**. Laboratory proficiency testing

*e.g., service delivery; health education programs; social marketing campaigns; program monitoring; electronic database construction and/or support; development of patient registries; needs assessments; and demonstration projects intended to assess organizational needs, management, and human resource requirements for implementation.

☐ **II. Activity is research but does NOT involve human subjects (Check one)**

- ☐ **A.** Activity is research involving collection or analysis of data about health facilities or other organizations or units (NOT persons).
- ☐ **B.** Activity is research involving data or specimens from deceased persons.
- ☐ **C.** Activity is research involving unlinked or anonymous data or specimens collected for another purpose.
- ☐ **D.** Activity is research involving data or specimens from animal subjects.§

§Note: Approval by CDC Institutional Animal Care and Use Committee (IACUC) may be required.

☐ **III. Activity is research involving human subjects but CDC involvement does not constitute "engagement in human subject research."** CDC employees or agents will not intervene or interact with living individuals or have access to identifiable information for research purposes. Appropriate IRB or ethics committee approval is required prior to approval.
(Check one)

- ☐ **A.** This project is funded under a grant/cooperative agreement/contract award mechanism.
- ☐ **B.** CDC staff provide technical support that does not involve possession or analysis of identifiable data or interaction with participants from whom data are being collected (No CDC Support[β]).
- ☐ **C.** CDC staff are involved only in manuscript writing for a project that has closed. For the project, CDC staff did not interact with participants and were not involved with data collection (No CDC Support).
- ☐ **D.** Activity is research involving linked data, but CDC non-disclosure form 0.1375B is signed.[∞]

β See definition of support on page 3.
∞ Access to linked data is permitted under any of the above sub-categories if CDC investigators and the holder of the key linking the data to identifiable human subjects enter into an agreement using CDC form 0.1375B, prohibiting the release of the key to CDC investigators under any circumstances. The purposes of the planned research do not contradict the terms of consent under which the information or specimens were collected, whether that consent was documented or not documented.

☐ **IV. Activity is research involving human subjects that requires submission to CDC Human Research Protection Office (Check one)**[α]

- ☐ A. Full Board Review (Use forms 0.1250, 0.1370-research partners)
- ☐ B. Expedited Review (Use same forms as A above)
- ☐ C. Exemption Request** (Use forms 0.1250X, 0.1370-research partners)
- ☐ D. Reliance[¥]
  - ☐ 1. Request to allow CDC to rely on a non-CDC IRB (Use same forms as A above, plus 0.1371)
  - ☐ 2. Request to allow outside institution to rely on CDC IRB (Use same forms as A above, plus 0.1372)

α There are other types of requests not listed under category IV, e.g., continuation of existing protocol, amendment, incident reports.
¥ Exemption and reliance request is approved by CDC Human Research Protection Office (HRPO).

CGH HSR Tracking #: 2016-0101

Amendment: If this request is an amendment to an existing project determination. Please include a brief description of the substantive change or modification below and attach both clean and marked copies of the amended protocol or project outline.

Submission: Attach a protocol or project description (See standard format below) in enough detail to justify the proposed category. Submit your request to your branch chief (or country director for DGHA country staff).

Approval Chain
Investigator → Branch Chief/Country Director →Division ADS → CGH Human Subjects Mailbox

**CGH ADS/ADLS Review**	**Date received in CGH ADS /ADLS office:**

☑ **Project does not require human subject research review beyond CGH at this time.**

☐ **Project constitutes human subject research that must be routed to CDC HRPO.**

Comments/Rationale for Determination:

**Approvals/Signatures:**	**Date:**	**Remarks:**
[signature] Digitally signed by Saleena Subaiya DN: cn=Saleena Subaiya, o, ou, email=yzv3@cdc.gov, c=US Date: 2016.04.18 16:40:14 -04'00'<br>Investigator	04/25/2016	
SHEFER.ABIGAIL.M.1029690410 Digitally signed by SHEFER.ABIGAIL.M.1029690410 DN: c=US, o=U.S. Government, ou=DoD, ou=PKI, ou=USPHS, cn=SHEFER.ABIGAIL.M.1029690410 Date: 2016.04.24 11:07:00 -04'00'<br>Branch Chief/Country Director	04/25/2016	
SHEFER.ABIGAIL.M.1029690410 Digitally signed by SHEFER.ABIGAIL.M.1029690410 DN: c=US, o=U.S. Government, ou=DoD, ou=PKI, ou=USPHS, cn=SHEFER.ABIGAIL.M.1029690410 Date: 2016.04.25 15:55:28 -04'00'<br>Division Human Research Protection Coordinator<br>Division ADS/ADLS or Director	04/25/2016	
[signature] Digitally signed by Aun Lor -S DN: c=US, o=U.S. Government, ou=HHS, ou=CDC, ou=People, cn=Aun Lor -S, 0.9.2342.19200300.100.1.1=1000001188 Date: 2016.05.04 08:15:33 -04'00'<br>CGH Human Research Protection Coordinator<br>CGH ADS/ADLS or Deputy ADS/ADLS	05/04/2016	

Note: Although CDC IRB review is not required for certain projects (categories I,II & III) approved under this determination, CDC investigators and project officers are expected to adhere to the highest ethical standards of conduct and to respect and protect to the extent possible the privacy, confidentiality, and autonomy of participants. All applicable country, state, and federal laws must be followed. Informed consent may be appropriate and should address all applicable elements of informed consent. CDC investigators should incorporate diverse perspectives that respect the values, beliefs, and cultures of the people in the country, state, and community in which they work.

Definitions

Agent – A nonemployee of CDC who conducts research under CDC's FWA. This generally includes all persons cleared for access to CDC networks and who use CDC networks or physical facilities for human research activities.

Epidemic disease control (aka, emergency response) – A public health activity undertaken in an urgent or emergency situation, usually because of an identified or suspected imminent health threat to the population, but sometimes because the public and/or government authorities perceive an imminent threat that demands immediate action. The primary purpose of the activity is to document the existence and magnitude of a public health problem in the community and to implement appropriate measures to address the problem (Langmuir, Public Health Reports 1980; 95:470-7).

Engagement – An institution becomes engaged in human subjects research when its employees or agents (i) obtain data about living individuals through intervention or interaction with them for research purposes; (ii) obtain individually identifiable private information about living individuals for research purposes; or (iii) obtain the informed consent of human subjects (http://www.hhs.gov/ohrp/FWAfaq.html). Furthermore, an institution is automatically considered to be engaged in human subjects research whenever it receives a direct HHS award to support such research, even where all activities involving human subjects are carried out by a subcontractor or collaborator.

Human subject or participant – is defined as a living person about whom an investigator conducting research obtains (1) data through intervention or interaction with the individual, or (2) identifiable private information (e.g., medical records, employment records, or school records).

Private information includes information about behavior that occurs in a context in which an individual can reasonably expect that no observation or recording is taking place, and information which has been provided for specific purposes by an individual and which the individual can reasonably expect will not be made public (for example, a medical record). Private information must be individually identifiable (i.e., the identity of the subject is or may readily be ascertained by the investigator or associated with the information) in order for obtaining the information to constitute research involving human subjects.

Program evaluation is the systematic collection of information about the activities, characteristics, and outcomes of programs to make judgments about the program, improve program effectiveness, and/or inform decisions about future program development. Program evaluation should not be confused with treatment efficacy which measures how well a treatment achieves its goals which can be considered as research. CDC guidance on research/non-research

Research – is defined as a systematic investigation, including research development, testing and evaluation, designed to develop or contribute to generalizable knowledge. Activities which meet this definition constitute research, whether or not these activities are conducted or supported under a program which is considered research for other purposes. For example, some demonstration and service programs may include research activities.

Support - Pertaining to Federal agencies, provision of funding, identifiable private information, or supplies, products, drug, other tangible support (does not include mere provision of Federal staff time and assistance absent other forms of financial or material support).

Surveillance – The ongoing systematic collection, analysis and interpretation of health data, essential to the planning, implementation and evaluation of public health practice, closely integrated to the dissemination of these data to those who need to know and linked to prevention and control.

Table: Determining Public Health Research from Nonresearch±

	Research	Practice (nonresearch)
**Definition**	"...systematic investigation, including research development, testing, and evaluation, **designed** to develop or contribute to generalizable knowledge." (ref. 45 CFR 46)<br><br>The purpose of the activity is to develop or contribute to generalizable knowledge to improve public health practice; intended benefits of the project can include study participants, but always extend beyond the study participants, usually to society; and data collected exceed requirements for care of the study participants or extend beyond the scope of the activity.	The purpose of the activity is to identify and control a health problem or improve a public health program or service; intended benefits of the project are primarily or exclusively for the participants (or clients) or the participants' community; data collected are needed to assess or improve the program or service, the health of the participants or the participants' community; knowledge that is generated does not extend beyond the scope of the activity; and project activities are not experimental. May use scientific methods to identify and control a health problem with benefits for the study participants or their communities.
**Primary Purpose**	To generate new or generalizable knowledge (information that can be applied in other settings)	To benefit study participants or the communities from which they come
**Methodology**	Scientific principles and methods used<br><br>Hypothesis testing/generating<br><br>Knowledge is generalizable	Scientific principles and methods may be used.<br><br>Hypothesis testing/generating<br><br>Knowledge may be generalizable
**Examples**		
**Surveillance Projects**	Requested data are broad in scope (and may involve as yet unproven risk factors)<br><br>Comparison of different surveillance approaches<br><br>Hypothesis testing<br><br>Subsequent studies planned using cases identified	Regular, ongoing collection and analyses to measure occurrence of health problem<br><br>Scope of data is health condition or disease, demographics, and known risk factors<br><br>Invokes public health mechanisms to prevent or control disease or injury
**Emergency Response**	Samples stored for future use<br><br>Additional analyses performed beyond immediate problem<br><br>Investigational drugs tested	Solves an immediate health problem<br><br>No testing of methods or interventions
**Program Evaluation**	Test an untried intervention<br><br>Systematic comparison of standard and nonstandard interventions, in any combination	Assess success of established intervention<br><br>Evaluation information used for feedback into program (management)

± Adapted from 2010 CDC Policy on "Distinguishing Public Health Research and Public Health Nonresearch"

Quick Reference Guide for Development of Scientific Protocol for Project Determination and Approval

Purpose

Investigators have responsibilities to provide complete and accurate information in order for reviewers to make informed-decision regarding the research/nonresearch and requirements for IRB review/approval. **The general rule of thumb is to include all available supporting documents for review when submitting a project for research/nonresearch determination.** If a protocol already exists, submit the protocol instead of a summary. Summary of protocol may omit crucial information. Additionally, all supporting documents, such as local institutional review board (IRB) approval and/or a letter of local ministry of health (MOH) concurrence, study tools, and informed consent documents should be submitted with the protocol for review. Protocol and all supporting documents must be in **English.** Investigators should discuss study issues with co-investigators, division ADSs, and other relevant partners prior to submission in order to determine appropriate category and subcategory and avoid delay in processing their request.

If a protocol has not already been written, the following outline, based on the CDC Protocol Development Guide, provides the minimal information needed for reviewer to make an informed-decision and assess the scientific and ethical merits of a study. Note that if the study is deemed human subjects research requiring IRB approval, a more detailed protocol may be requested for submission to the IRB. CDC Excellence in Science Committee has developed a protocol development guide that should be used in preparing protocols for submission to the IRB. The guide can be found at http://www.cdc.gov/niosh/nas/mining/pdfs/Protocol%20Checklist.pdf.

General Outline

I. Overview

- ***Title:*** A project title should provide the main idea of the study and include country name(s). Be specific and avoid general title that tells little about the actual study.
- ***Project summary:*** Give a concise overview of the project. Describe the purpose of the study, including problem to be investigated and hypothesis(es) to be tested, the population, and the methods that will be used. Avoid the use of acronyms. Include the expected benefit of the study.
- ***Investigators/collaborators/funding mechanism(s)/Federalwide Assurance numbers:*** Include the names and degrees of all investigators (CDC and collaborators); include SEV# for CDC investigators, their roles, responsibilities, and interaction with participants in the project. Include the following information for each collaborator: funding mechanism, award number, FederalWide Assurance number if applicable.
- **OMB/PRA information:** Describe the proposed information collection, how the information will be collected, by whom, and what role CDC staff member will have. Note that any standardize data collection on 10 or more people where CDC activities include approving, directing, managing, and owning the data will require OMB/PRA determination.
- **Sponsoring institution(s):** Identify the sponsoring institution(s) if not CDC (Note that if this is blank, it is assumed that CDC is the sponsoring institution).

II. Introduction/Background

- ***Literature review/current state of knowledge about project topic:*** Discuss relevant information about the subject of the project based on a review of the literature. Provide citation of the sources and include a reference in the appendix.
- ***Justification for study:*** Explain the public health and scientific importance of the study. In the context of previous studies, describe the contribution this study will make.
- ***Location(s):*** Identify the study location(s).

- ***Intended/potential use of study findings:*** Define the primary target audience(s) and discuss the expected applicability of study findings.
- ***Goals/Objectives:*** Clearly and concisely list the goals and/or objectives that the project will address.
- ***Hypotheses or questions:*** Describe the question(s) that the study will answer**.** State the type of hypothesis(es) that will be explored or tested.

III. Methods

- ***Study design/timeline:*** Describe the methods to be used, the duration of participants' involvement. Describe whether the approach used will be descriptive, exploratory (hypothesis-generating), confirmatory (hypothesis-testing), or developmental (focused on corrective action).
- ***Study population(s):*** Describe the study population, number of participants, sampling frame, case definitions, inclusion/exclusion criteria, recruitment, and justification for involving vulnerable populations.
- ***Study procedures:*** Describe training, number of staff and roles, data collection activities, adverse event/protocol deviation reporting, monitoring plan, etc.
- ***Data analysis:*** Describe data management, storage, quality assurance, and key variables that will be collected and how data will be analyzed.
- ***Dissemination/Reporting of Results:*** Describe any plan for notifying participants and other stakeholders of study findings. Describe any publication plan.

IV. *Ethical considerations*:

- ***Risks/Benefits:*** Describe the potential risks (physical and mental) and benefits to study participants
- ***Informed Consent/Assent/Permission:*** Describe the informed consent/assent procedures, waiver of informed or written consent, assent of children, parental permission,
- ***Other ethical considerations:*** Describe confidentiality/privacy protection, autonomy, safeguard for vulnerable population, reporting of adverse events, and culture, values, and beliefs. Describe conflicts or potential conflicts of interest if any.
- ***HIV Notification Policy:*** If individuals will be consented and tested for HIV, describe the plan to inform individuals of their test results, including providing appropriate counseling according to HHS Policy -- http://www.hhs.gov/ohrp/policy/hsdc88jun.html.

References: List of references cited.
Appendices: Include all relevant materials, such as study tools, informed consent document, local IRB approval, confidentiality agreement, material/data transfer agreement, and other supporting documents.

Links

- CDC Human Research Protections Policy (2010): http://aops-mas-iis.cdc.gov/Policy/Doc/policy556.pdf
- CDC Distinguishing Public Health Research and Public Health Nonresearch (2010): http://aops-mas-iis.cdc.gov/policy/Doc/policy557.pdf
- CDC Scientific Ethics Verification # database (intranet): http://inside.od.cdc.gov/scientificethics/reprintmenu.asp
- HHS Title 45 Code of Federal Regulations Part 46, Protection of Human Subjects (Revised 2009): http://www.hhs.gov/ohrp/humansubjects/guidance/45cfr46.html
- OHRP Guidance on Engagement of Institutions in Human Subjects Research: http://www.hhs.gov/ohrp/policy/engage08.html
- OHRP Guidance on Research Involving Coded Private Information or Biological Specimens: http://www.hhs.gov/ohrp/policy/cdebiol.html
- OHRP FederalWide Assurance number database: http://ohrp.cit.nih.gov/search/search.aspx?styp=bsc
