# Supplementary material for: Use of the revised World Health Organization cluster survey methodology to classify measles-rubella vaccination campaign coverage in 47 counties in Kenya, 2016
Source: PLoS One. 2018 Jul 2;13(7):e0199786. doi: 10.1371/journal.pone.0199786 (PMC6028100; doi:10.1371/journal.pone.0199786)
Supplement: S3 File — (PDF) [file pone.0199786.s003.pdf]

Household Consent and Eligibility

Kenya Measles-Rubella Post-Campaign Coverage Survey Questionnaire

Nambari ya Kikundi ___ ___ ___ ___
Cluster Number

Jina la Kikundi______________________
Cluster name

Kaunti ___________________________
County

Nambari ya kaunti ___ ___ ___
County Number

Wilaya _____________________________
District

Tarafa ______________________________
Division

Kata _______________________________
Location

Kata ndogo____________________________
Sub-location

Mashambani=1/ Mjini=2 ________________________________
Rural=1/ Urban= 2

Nambari ya Msimamizi ___ ___ ___ ___
Supervisor Number

Jina la Msimamizi ___________________________________
Supervisor Name

Nambari ya anayehoji ___ ___ ___ ___
Interviewer Number

Jina la anayehoji ____________________________________
Interviewer Name

Nambari ya nyumba ___ ___ ___ ___ - ___ ___ ___
Household Serial Number (Cluster number) (HH number) *[Record at top of every page]*

[If someone is home, please read consent. If no one is home, dwelling not found, or dwelling is vacant, skip consent and fill out interview visit table.]

"Habari, jina langu ni ________________, ninafanya na wizara ya Afya tukishirikiana na Shirika La Takwimu la Kenya. Tunafanya utafiti nchini kote kuhusu kampeni ya chanjo ya Ukambi na Rubella. Tungependa kukuuliza maswali kuhusu familia yako na hali ya chanjo ya Ukambi kwa watoto katika familia yako. Ujumbe huu utasaidia Wizara ya Afya kuboresha huduma za chanjo kote nchini. Maswali yote yatachukua kati ya dakika 20-25 kukamilika. Kujibu maswali haya ni kwa ihiari yako na unaweza kukataa kushiriki. Ujumbe wowote utakaotupa utakuwa wa siri na hautatolewa kwa mtu yeyote mwingine."

"Hello, My name is__________________ and am working with the Ministry of Health in collaboration with the Kenya National Bureau of Statistics. We are conducting a survey across the country about measles and rubella vaccination. We would like to ask some questions about your household and the measles vaccination status of children in your household. This information will help the Ministry of Health to improve the immunization services in the country. The questionnaire usually takes about 20-25 minutes to complete. Participation is voluntary and you can stop at any time. You can refuse to participate without any negative result. Whatever information you provide will be kept strictly confidential."

Household serial number ___ ___ ___ ___–___ ___ ___

“Naweza kukuuliza maswali sasa?
“May I begin the Interview now?”

- ☐ Yes, permission is given. ⇨ **Continue to next question.**

- ☐ If permission is not given, stop the interview. ⇨ Record refusal on the interviewer visit table and re-use this same questionnaire for the next eligible household.

“ Kuna watoto waliolala ndani ya nyumba hii usiku wa kuamkia leo ambao wana umri kati ya miezi tisa na miaka kumi na nne?”
“Are there any children between 9 months and 14 years of age who slept here last night?”

- ☐ Yes, have eligible child. ⇨ **Continue to interviewer visit table and then proceed to Coverage Survey Questionnaire**

- ☐ If no, the interview is over. ⇨ Record household is not eligible on interviewer visit table below.

Interviewer Visit Table

[Record date and time of first visit, and final status code if assigned. If the mother/caretaker of an eligible child is not home, attempt a second visit and third visit if necessary and record the time.]

	**1**	**2**	**Mahojiano ya mwisho**
Tarehe (Tarehe/mwezi/mwaka)<br><br>Nambari *:	_____/_____/_______<br>☐	_____/_____/_______<br>☐	_____/_____/_______<br>☐
Siku ya mahojiano ijayo<br>Tarehe :<br><br>Saa :	_____/_____/_______<br>____________________	_____/_____/_______<br>____________________	

*Status code: **1. Nimekamilisha mahojiano**/Consented to interview and eligible **2. Hakuna mtoto wa miezi 9 hadi miaka 14 hapa**/ No eligible age children **3. Nimehairisha mahojiano** (rudi)/ Postponed (return) **4. Hakuna mtu mzima kwa nyumba (rudi)** / No adult at home (return) **5. Watu wote hawako nyumbani kwa muda mrefu** / Entire household absent for extended period of time **6. Kukataa** /Refused **7. Boma halipatikani** / Dwelling not found **8. Wanaoishi hawako** / Dwelling vacant/ Does not exist **9. Nyingine- (Taja)** / Other- (Specify)

Household serial number ___ ___ ___ ___–___ ___ ___

Kenya Measles-Rubella Post-Campaign Coverage Survey
Household Questionnaire

“Kwanza ningependa kukuulizia kuhusu wanaoishi ndani ya nyumba hii.”
“First, I’d like to ask about the members of this household.”

Jina la mkuu wa nyumba ________________________
Name of household head

Je, ni watu wangapi wanaishi ndani ya nyumba hii?
How many persons live in the household?

Idadi/ Number _________

Ni watoto wangapi walilala hapa usiku wa kuamkia leo? Tafadhali orodhesha watoto hao, pamoja na siku ya kuzaliwa, umri kwenye mpangilio ufuatao. (***Watoto wanaostahili ni wale walio na umri kati ya miezi 9 na miaka 14, au wale waliozaliwa kati ya 17 May 2001 na 24 Agosti 2015***)
How many children slept here last night? Please list them, their birthdates, and ages in the table below.
*(All eligible children will be those aged **9months-14 years**, or those born between **17 May 2001- 24 Aug. 2015)***

[Child eligibility table]

**Nambari** Number	**Jina la Kwanza la mtoto** Child’s First Name	**Tarehe ya kuzaliwa** [_ _/ _ _/ _ _ _ _] TT/Mwezi/Mwaka *[Ikiwa tarehe ya kuzaliwa haipatikani, jaza **99**]* Date of birth [DD/MM/YYYY] *[If unavailable, record 99]*	**Miaka ya mtoto tarehe 24 Mei 2016** *[Ikiwa hajafikisha mwaka mmoja, jaza kwa miezi, ikiwa zaidi ya mwaka moja, jaza miaka]* Age of child on **24 May 2016?** *[If less than 1 year, write in months. If more than 1 year, write in years]*		**Anafaa (Ndio,La)** Eligible (Y, N)
			**Miaka** (Years)	**Miezi** (Months)	
1					
2					
3					
4					
5					
6					
7					
8					
9					
10					
11					
12					
13					
14					
15					

Kuna akina mama (walezi) wangapi wenye watoto wa umri wa miezi 9 hadi miaka 14 waliolala hapa usiku wa kuamkia leo? [Uliza: Je, kuna akina mama wengine wanaoishi ndani ya nyumba na watoto wenye umri huu? Kwa mfano, bibi wengine, shangazi, na wasaidizi wa nyumbani?]

Household serial number ___ ___ ___ ___–___ ___ ___

How many mothers (or primary caregivers) are there for the children listed above who are aged 9 months to 14 years who slept here last night? [Prompt: are there any other women staying in the household with children this age, like other wives, aunts and house help?]

Idadi ya akina mama/walezi ____________________
Number of mothers/caregivers

[Mother/caregiver table]

[Please list all mothers or primary caregivers of children aged 9 months-14 years of age who slept in the household last night, including those not present at the time of the survey. Use the child table from the previous page to determine the number of eligible children each mother has]

**Nambari** Number	**Jina la mama au Mlezi wa mtoto** Name of mother/caregiver	**Nambari ya watoto kati ya miezi 9 na miaka 14** Number of children aged 9 months-14 years	**Umri ya mama** Age of mother
**1**			
**2**			
**3**			
**4**			
**5**			
**6**			

[Selecting a respondent]

[If there is more than one mother/caregiver with eligible children, select one using the random number table. To use it, circle the total number of eligible mothers/caregivers in the column on the top. Then, circle the final numeral of the household number in the row on the left. Circle the box where this column and row intersect. That number will be your randomly chosen number to identify the mother listed in Table 1.]

**Random Number Table**		**Number of Eligible Mothers/Caregivers**				
		**2**	**3**	**4**	**5**	**6**
**Last digit of Household Number**	**0**	**2**	**3**	**4**	**1**	**5**
	**1**	**2**	**1**	**2**	**1**	**2**
	**2**	**2**	**1**	**4**	**2**	**3**
	**3**	**1**	**3**	**3**	**3**	**4**
	**4**	**2**	**1**	**2**	**5**	**4**
	**5**	**2**	**1**	**2**	**3**	**5**
	**6**	**1**	**2**	**4**	**4**	**1**
	**7**	**2**	**3**	**3**	**5**	**2**
	**8**	**1**	**2**	**2**	**3**	**6**
	**9**	**1**	**3**	**4**	**4**	**1**

Household serial number ___ ___ ___ ___–___ ___ ___

**Sehemu ya A: Fomu ya kukubali na kuchaguliwa kushiriki mahojiano** / Section A: Consent and Eligibility	
A1	**[Jaza nambari ya mama/Mlezi aliyechaguliwa hapa. Ikiwa mama/Mlezi ni mmoja, jaza 1]**<br>[Write number and name of selected mother/caregiver here. If only one mother/caregiver, write '1']<br><br>**Nambari** / Number ____________________ **Jina**/ Name ___________________________
A2	**Je, mama/Mlezi aliyechaguliwa anapatikana kwa sasa?**<br>Is the selected mother/caregiver available now?<br><br>☐ 1. Ndio [enda **A4**] — 1. Yes [Skip to **A4**]<br>☐ 2. La — 2. No
A3	**Je, kuna wakati mwingine ninaweza kurudi na kuzungumza naye ama je kuna watu wazima ambao wangeweza kujibu maswali kuhusu afya ya watoto wao?**<br>Is there a time when I could return and talk to her/him? Or is there another adult who could answer questions about the healthcare information of her/his children?<br><br>☐ 1. Rudi baadaye [kumbuka kuahirishwa katika kijikaratasi ya kuwasajili vikundi na rudi watakapopatikana] — 1. Return later [Note "postponed" on interview visit table and return when available]<br>☐ 2. Mhoji mtu mwingine — 2. Use another respondent
A4	**Je, kuna uhusiano gani kati ya anayehojiwa na mtoto?**<br>What is the respondent's relationship to child?<br><br>☐ 1. Mama — 1. Mother<br>☐ 2. Baba — 2. Father<br>☐ 3. Ndugu/dada — 3. Sibling<br>☐ 4. Nyanya — 4. Grandmother<br>☐ 5. Shangazi — 5. Aunt<br>☐ 6. Mfanyikazi wa nyumba — 6. House help/maid<br>☐ 7. Mwingine (eleza) _________ — 7. Other (specify) _______________

**Sehemu ya B: Kujua kuhusu kampeni ya chanjo ya ukambi na rubella**/ Section B: Measles-rubella vaccination campaign awareness	
B1	**Je, unajua kuhusu kampeni ya chanjo ya ukambi na rubella iliyofanyika mwezi uliopita?**<br>Were you aware of the measles-rubella vaccination campaign occurring last month?<br>☐ 1. Ndio — 1. Yes<br>☐ 2. La [enda **C1**] — 2. No [Skip to **C1**]
B2	**Je ulipata ujumbe kuhusu kampeni hiyo kutoka wapi? (Chagua ya kwanza kama njia muhimu ya kupata habari)**<br>What is your main source of information regarding the campaign? [Choose the first source that the mother reports as MAIN source]<br>☐ 1. Mhudumu wa afya — 1. Health worker<br>☐ 2. Mgeni — 2. House visitor<br>☐ 3. Kipaza sauti/ tangazo kwa umma — 3. Loudspeaker/ Public announcement<br>☐ 4. Mabano — 4. Poster/banner<br>☐ 5. Gazeti — 5. Newspaper<br>☐ 6. Redio — 6. Radio<br>☐ 7. Televisheni — 7. Television<br>☐ 8. Simu ya mkono/ujumbe mfupi — 8. Mobile phone/SMS<br>☐ 9. Familia/Jirani/Rafiki — 9. Family/neighbor/friends<br>☐ 10. Kikundi (Cha akina mama /vijana) — 10. Women's/Youth group<br>☐ 11. Kanisani/ Msikitini — 11. Church/mosque/temple<br>☐ 12. Kiongozi wa jamii — 12. Community leader<br>☐ 13. Waliopeana chanjo — 13. Campaign vaccinator<br>☐ 14. Mtandao — 14. Internet/social media<br>☐ 15. Ingine (Eleza) ______________ — 15. Other (Specify) ______________
B3	**Tafadhali, orodhesha njia zingine za kupata habari kuhusu kampeni**<br>Please list all other sources of information regarding the campaign<br>☐ 1. Mhudumu wa afya — 1. Health worker<br>☐ 2. Mgeni — 2. House visitor<br>☐ 3. Kipaza sauti/ tangazo kwa umma — 3. Loudspeaker/ Public announcement<br>☐ 4. Mabano — 4. Poster/banner<br>☐ 5. Gazeti — 5. Newspaper<br>☐ 6. Redio — 6. Radio<br>☐ 7. Televisheni — 7. Television<br>☐ 8. Simu ya mkono/ujumbe mfupi — 8. Mobile phone/SMS<br>☐ 9. Familia/Jirani/Rafiki — 9. Family/neighbor/friends<br>☐ 10. Kikundi (Cha akina mama/vijana) — 10. Women's/Youth group<br>☐ 11. Kanisani/ Msikitini/ — 11. Church/mosque/temple<br>☐ 12. Kiongozi wa jamii — 12. Community leader<br>☐ 13. Waliopeana chanjo — 13. Campaign vaccinator<br>☐ 14. Mtandao — 14. Internet/social media<br>☐ 15. Ingine (Eleza) ______________ — 15. Other (Specify) ______________
**"Sasa nitakuuliza kuhusu watoto wako na kama wamewahi kupata chanjo aina yoyote** "<br>"Next, I'm going to ask about the children and their vaccination histories."	

Household serial number ___ ___ ___ ___–___ ___ ___

Sehemu ya C: Kampeni ya Chanjo dhidi ya ukambi na rubella ya watoto wenye umri wa miezi 9 hadi miaka 14/ Section C: MR Campaign Vaccination for Children Aged 9 months – 14 years

**Nambari ya mtoto**<br><br>Number of child	**C1**	**C2**	**C3**		**C4**	**C5**	**C6**	**C7**
	**Jina la mtoto (Miezi 9 hadi miaka 14)**<br><br>Name of child *[Aged 9 months to 14 years]*	**Tarehe ya kuzaliwa** [_ _/ _ _/ _ _ _ _] TT/Mwezi/Mwaka *[enda **C4**]* *[Ikiwa tarehe ya kuzaliwa haipatikani, jaza **99** kwenye nafasi hii, enda swali **C3**]*<br><br>Date of birth [DD/MM/YYYY] *[Skip to **C4**]* *[If unavailable, record 99 and proceed to question **C3**]*	**Miaka ya mtoto tarehe 24 Mei 2016** *Ikiwa hajafikisha mwaka mmoja, jaza kwa miezi, ikiwa ana zaidi ya mwaka mmoja, jaza miaka]*<br><br>Age of child on 24 May 2016? *[If less than 1 year, write in months. If more than 1 year, write in years]*		**Jinsia ya mtoto Kiume=1 Kike=2**<br><br>Sex of Child Male=1 Female =2	**Orodha ya kuzaliwa** 1, 2,3…*n.k* ***(Ikiwa orodha haijulikani au mtoto ni wakuigwa jaza 99)***<br><br>Birth order 1,2,3… etc *[If birth order unknown, or child adopted, write 99]*	**Je, mtoto anaenda shule?** Shule ya mchana=1 Shule ya Bweni=2 La =3 Sijui=4<br><br>Is the child going to school? Day school=1 Boarding school=2 No=3 Don't know=4	**Je, mtoto yuko hapo wakati wa mahojiano?** Ndio=1 La=2<br><br>Is the child present at the time of the interview? Yes=1 No=2
			Years (Miaka)	Months (Miezi)				
**1**								
**2**								
**3**								
**4**								
**5**								
**6**								
**7**								
**8**								

CHILD Questionnaire No. ____ OF _____ (Complete additional forms for more than 8 children. Please indicate how many CHILD Questionnaires are completed.
<u>Keep CHILD ID of the surveyed children fixed on each page. If ID of a child James is 03 on page 1, it will remain 03 on all pages</u>

Household serial number ___ ___ ___ ___–___ ___ ___

Sehemu ya C: Kampeni ya Chanjo dhidi ya ukambi na rubella ya watoto wenye umri wa miezi 9 hadi miaka 14/ Section C: MR Campaign Vaccination for Children Aged 9 months – 14 years

	C8	C9	C10
**Nambari ya mtoto**<br><br>Number of child	**Je, mtoto alipata chanjo ya ukambi-rubella wakati wa kampeni**<br>*[Jibu moja – dhibitisha kwa kutizama wino kwenye kidole au ujumbe kutoka kwa mhudumu] (ona vielezo) [ikiwa ni la, enda* ***C10****]*<br><br>Did the child receive the measles-rubella vaccine during the campaign?<br>*[ONE* response- *Fingermark preferred to recall]* (*see code) [If* ***N****, skip to* ***C10****]*	**Mtoto alipata chanjo ya ukambi-rubella katika eneo gani/wapi?**<br>(ona vielezo) ***[enda C11]***<br><br>At what location did your child receive the measles-rubella vaccine?<br>*(see code) [Skip to* ***C11****]*	**Je, ni sababu gani kuu ilizuia mtoto kupata chanjo wakati wa kampeni?**<br>*[Jibu moja- sababu ya kwanza itakayotajwa na]*<br>*(ona vielezo)*<br><br>What was the main reason for the child not receiving the campaign vaccine?<br>*[ONE response- note the FIRST REASON mentioned by the respondent]*<br>*(see code)*
**1**			
**2**			
**3**			
**4**			
**5**			
**6**			
**7**			
**8**			

CHILD Questionnaire No. ____ OF _____ (Complete additional forms for more than 8 children. Please indicate how many CHILD Questionnaires are completed.
Keep CHILD ID of the surveyed children fixed on each page. If ID of a child James is 03 on page 1, it will remain 03 on all pages.

Household serial number ___ ___ ___ ___–___ ___ ___

Sehemu ya C: Kampeni ya Chanjo dhidi ya ukambi na rubella ya watoto wenye umri wa miezi 9 hadi miaka 14/ Section C: MR Campaign Vaccination for Children Aged 9 months – 14 years

CODE (Do not write on here, fill in answer box on previous sheet using codes below)

C8		C9		C10	
**Ndio, kuna wino kwenye kidole**<br>Yes, fingermark present	1	**Shule** / School	1	**Kutojua kuhusu kampeni** / Unaware of campaign	01
				**Kutojua umuhimu wa chanjo** / Unaware of need for vaccination	02
		**Hospitali ya Serikal**<br>Govt. Health facility	2	**Mtoto hakuwepo wakati wa kampeni ya chanjo** / Child absent during vaccination campaign	03
**Ndio, ujumbe kutoka kwa Mlezi**<br>Yes, by recall of caretaker	2			**Mtoto tayari amechanjwa** / Child already vaccinated	04
		**Hospitali ya kibinafsi**<br>Private Health facility	3	**Mtoto alikuwa mgonjwa** / Child sick	05
				**Sikuwa na wakati wa kwenda kwenye kampeni** /Too busy to go to campaign	06
				**Nilisahau kumpeleka mtoto kwenye kampeni ya chanjo** / Forgot to take child for vaccination campaign	07
**La** / No	3	**Sokoni** / Market	4	**Pahali pa kutolewa chanjo ni mbali** /Post too far	08
**Sijui** / Don't know	4	**Kwenye kijiji**<br>Village meeting point	5	**Nilienda lakini laini ilikuwa ndefu sikusubiri** / Went, but line too long so didn't wait	09
				**Nilienda lakini chanjo haikuwa hospitalini** / Went, but vaccine not available at facility	10
				**Nilienda lakini kituo cha kupeana chanjo kilikuwa kimefungwa**/ Went, but vaccination post/clinic closed	11
		**Kanisani/Msikitini**<br>Church/Mosque	6	**Mhudumu wa afya aliniongelesha vibaya** / Health staff rude	12
				**Mama alikataa** / Mother refused	13
		**Pahali pa kupokea Chanjo**<br>Temporary fixed vaccination post	7	**Mume/mkuu wa nyumba alikataa** / Husband/head of household refused	14
				**Naamini kuwa chanjo inaweza kudhuru** / Believe that vaccine can cause harm	15
				**Siamini chanjo** / Do not trust vaccines	16
				**Sababu za kidini/kitamaduni** / Religious/cultural reasons	17
		**Nyumbani** / House	8	**Chanjo ya kampeni sio nzuri kama ile ya klinik/hospitali** / Campaign vaccine not as good as vaccine offered through routine health services	18
				**Sijui** / Don't know	19
		**Pahali pengine, (Elezea)**<br>Other (Specify)	9	**Sababu zinigine (Elezea)** / Other (Specify)	20

Household serial number ___ ___ ___ ___–___ ___ ___

Sehemu ya C: Kampeni ya Chanjo dhidi ya ukambi na rubella ya watoto wenye umri wa miezi 9 hadi miaka 14/ Section C: MR Campaign Vaccination for Children Aged 9 months – 14 years

	**C11**	**C12**	**C13**	**C14**	**C15**	**C16**	**C17**
**Nambari ya mtoto**<br><br>Number of child	**Sasa nitakuuliza maswali kuhusu chanjo ya kawaida kwa mtoto wako**<br>**Je, mtoto ashawahipata hata chanjo moja ya ukambi wakati wa kliniki**<br>*Jibu moja, kadi ni rahisi kukumbuka*<br>*[kama la enda **C15**]*<br><br>Now I am going to ask you questions about your child's routine immunizations.<br>Did the child receive at least one dose of measles vaccine through routine health services?<br>*[ONE response- Order of preference: Card preferred to recall]*<br>*[IF N, go to **C15**]*	**Kama tarehe imeandikwa kwenye kadi ya kliniki, andika tarehe ya chanjo ya kwanza ya ukambi.**<br>[_ _/ _ _/ _ _ _ _]<br>(TT/Mwezi/Mwaka)<br>[Ikiwa tarehe haiko andika **99**]<br><br>If date legible on vaccination card, give the date of *first* routine measles vaccination.<br>[_ _/ _ _/ _ _ _ _]<br>(DD/MM/YYYY)<br>*[If date unavailable, record 99 here]*	**Je, mtoto alipata chanjo ya pili ya ukambi?**<br>Ndio=1<br>La=2<br>Sijui=3<br>[Ikiwa ni La, enda **C16**]<br><br>Did the child receive a *second* routine measles dose?<br>Yes=1<br>No=2<br>Don't know=3<br>*[IF N, go to **C16**]*	**Ikiwa tarehe imeandikwa kwenye kadi ya kliniki, andika siku ya chanjo ya pili ya ukambi.**<br>[_ _/ _ _/ _ _ _ _]<br>(TT/Mwezi/Mwaka)<br>[Ikiwa tarehe haiko andika **99**, kisha enda **C16**]<br><br>If date legible on vaccination card, give the date of *second* routine measles vaccination.<br>[_ _/ _ _/ _ _ _ _]<br>(DD/MM/YYYY)<br>*[If date unavailable, record 99 here and skip to **C16**]*	**Je, kwanini mtoto hakupata chanjo ya ya ukambi kwa kliniki?**<br>*[Jibu moja-andika sababu ya kwanza itakayotajwa.]*<br>*(Ona vielezo)*<br><br>Why did the child not receive a routine measles dose?<br>*[ONE response- note the FIRST REASON mentioned by the respondent]*<br>*(see code)*	**Je, mtoto alipata chanjo yoyote ya ukambi wakati wa kampeni zingine** [2012, 2009, 2006, 2002]<br>Ndio=1<br>La=2<br>Sijui=3<br><br>Did the child receive any measles doses during previous campaigns?<br>[2012, 2009, 2006, 2002]<br>Yes=1<br>No=2<br>Don't know=3	**Mtoto alipata chanjo mara ngapi wakati wa kampeni zilizopita?**<br>[1,2,3 au 4]<br><br>How many previous campaign doses did the child receive?<br>[1,2,3, or 4]
**1**							
**2**							
**3**							
**4**							
**5**							
**6**							
**7**							
**8**							

CHILD Questionnaire No. ____ OF _____ (Complete additional forms for more than 8 children. Please indicate how many CHILD Questionnaires are completed.

<u>Keep CHILD ID of the surveyed children fixed on each page. If ID of a child James is 03 on page 1, it will remain 03 on all pages.</u>

Household serial number ___ ___ ___ ___–___ ___ ___

Sehemu ya C: Kampeni ya Chanjo dhidi ya ukambi na rubella ya watoto wenye umri wa miezi 9 hadi miaka 14 / Section C: MR Campaign Vaccination for Children Aged 9 months – 14 years

CODE (Do not write on here, fill in answer box on previous sheet using codes below)

C11		C15	
**Ndio, kadi ya kliniki iko**<br>Yes, vaccination card or child booklet available	1	**Kutojua umuhimu wa chanjo** / Unaware of need for vaccination	01
		**Sikuwa na wakati wa kumpeleka kwa chanjo kwenye kliniki** /Too busy to take child in for routine vaccination	02
		**Nilisahau kumpeleka mtoto chanjo** / Forgot to take child for vaccinations	03
**Ndio,** ujumbe wa Mlezi<br>Yes, by recall of caretaker	2	**Mtoto alikuwa mgonjwa** / Child sick	04
		**Pahali pa kutolewa chanjo ni mbali** / Vaccination post too far	05
		**Nilienda lakini laini ilikuwa ndefu sikusubiri** / Went, but line too long so didn't wait	06
**La** / No	3	**Nilienda lakini chanjo haikuwa hospitalini** / Went, but vaccine not available at facility	07
		**Nilienda lakini kituo cha kupeana chanjo kilikuwa kimefungwa** / Went, but vaccination post/clinic closed	08
**Sijui** / Don't know	4	**Mhudumu wa afya aliniongelesha vibaya** / Health staff rude	09
		**Chanjo inaweza kudhuru** / Believe that vaccine can cause harm	10
		**Siamini chanjo** / Do not trust vaccines	11
		**Sababu za kidini/kitamaduni** / Religious/cultural reasons	12
		**Mama alikataa** / Mother refused	13
		**Mume/mkuu wa nyumba alikataa** / Husband/head of household refused	14
		**Sijui** / Don't know	15
		**Sababu zingine /** Other (Specify)	16

Household serial number ___ ___ ___ ___ –___ ___ ___

**Sehemu ya D: Matumizi ya simu ya mkono na ujumbe mfupi** / Section D: Mobile phone use	
**Sasa nitakuuliza kuhusu utumizi wako wa rununu (simu)**<br>"Next, I'm going to ask about mobile phone use."	
**D1**	**Je una rununu (simu)?**<br>Do you own a mobile phone?<br>☐ 1. Ndio *[enda D4]* — 1. Yes *[skip to D4]*<br>☐ 2. La — 2. No
**D2**	**Kunayo rununu (simu) kwenye nyumba ambayo unaweza tumia?**<br>Is there a mobile phone in the household that you use?<br>☐ 1. Ndio — 1. Yes<br>☐ 2. La *[enda D17]* — 2. No *[skip to D17]*
**D3**	**Rununu (simu) hiyo ni ya nani?**<br>Whose phone is it?<br>☐ 1. Familia — 1. Family<br>☐ 2. Rafiki — 2. Friend<br>☐ 3. Simu ya jamii — 3. Community phone<br>☐ 4. Ingine, eleza: ____________________________ — 4. Other, specify:_________________
**D4**	**Ni mtandao upi wa simu unatumia wakati mwingi? *[Chagua moja]***<br>Which mobile phone network do you use most of the time? *(Choose only one)*<br>☐ 1. Safaricom — 1. Safaricom<br>☐ 2. Airtel/Zain — 2. Airtel/Zain<br>☐ 3. Orange — 3. Orange<br>☐ 4. Yumobile — 4. Yumobile<br>☐ 5. Equitel — 5. Equitel<br>☐ 6. Ingine, eleza:__________________ — 6. Other, specify:____________________
**D5**	**Ni mara ngapi huwa una shida ya kupiga simu kwa sababu ya shida za mtandao (network)?**<br>How often do you have problems making phone calls because of poor network?<br>☐ 1. Hakuna — 1. Never<br>☐ 2. Nadra — 2. Rarely<br>☐ 3. Mara mwa mara — 3. Sometimes<br>☐ 4. Mara nyingi — 4. Frequently<br>☐ 5. Kila siku — 5. Daily<br>☐ 6. Sijui — 6. Don't know
**D6**	**Je, unatumia mtandao (network) mwingine wapili wa simu?**<br>Do you use any additional mobile phone networks?<br>☐ 1. Ndio — 1. Yes<br>☐ 2. La *[enda D8]* — 2. No *[skip to D8]*
**D7**	**Ikiwa ni ndio, mtandao (network) upi mwingine? (Chagua zote zilozotajwa)**<br>What are the other mobile networks you use? *[Check all that are mentioned]*<br>☐ 1. Safaricom — 1. Safaricom<br>☐ 2. Airtel/Zain — 2. Airtel/Zain<br>☐ 3. Orange — 3. Orange<br>☐ 4. Yumobile — 4. Yumobile<br>☐ 5. Equitel — 5. Equitel<br>☐ 6. Ingine, eleza:__________________ — 6. Other, specify:____________________
**D8**	**Je, wewe hutuma ujumbe mfupi (SMS)?**<br>Do you send text messages (SMS)?<br>☐ 1. Ndio — 1. Yes<br>☐ 2. La *[enda D11]* — 2. No *[skip to D11]*

**D9**	**Ikiwa ndio, unatuma ujumbe mfupi (SMS) mara ngapi?**<br>How frequently do you send text messages/SMS?<br>☐ 1. Nadra — 1. Rarely<br>☐ 2. Kila mwezi — 2. Monthly<br>☐ 3. Kila wiki — 3. Weekly<br>☐ 4. Kila siku — 4. Daily
**D10**	**Ni mara ngapi huwa una shida ya kutuma ujumbe mfupi (SMS) kutokana na shida za kukosa mtandao?**<br>How often do you have problems sending text messages/SMS because of poor network?<br>☐ 1. Nadra — 1. Rarely<br>☐ 2. Kila mwezi — 2. Monthly<br>☐ 3. Kila wiki — 3. Weekly<br>☐ 4. Kila siku — 4. Daily<br>☐ 5. Sijui — 5. Don't know
**D11**	**Je, huwa unasoma ujumbe mfupi (SMS) ya matangazo unazopokea?**<br>Do you read text message (SMS) advertisements that you receive?<br>☐ 1. Ndio — 1. Yes<br>☐ 2. Saa zingine — 2. Sometimes<br>☐ 3. La — 3. No
**D12**	**Je ulipokea ujumbe (SMS) kupitia Kwa simu kuhusu kampeni ya chanjo dhidi ya ukambi-rubella mwezi uliopita?**<br>Did you receive a text message (SMS) about the measles-rubella vaccination campaign in the last month?<br>☐ 1. Ndio — 1. Yes<br>☐ 2. La *[enda D17]* — 2. No *[skip to D17]*
**D13**	**Ulipokea ujumbe (SMS) ngapi kuhusu kampeni ya chanjo ya ukambi na rubella?**<br>How many text messages (SMS) about the measles-rubella vaccination campaign did you receive?<br>**Idadi /** Number _________
**D14**	**Je kwa maoni yako, nieleze kuhusu kupokea ujumbe mfupi (SMS) kuhusu kampeni ya chanjo ya ukambi-rubella?**<br>What was your opinion about receiving the text messages (SMS) about the measles-rubella vaccination campaign?<br>☐ 1. Mazuri (walipenda / zilisaidia) — 1. Favorable (liked it/helpful)<br>☐ 2. Hakuna maoni — 2. Neutral or no opinion<br>☐ 3. Mbaya (hawakupenda/ waliudhika) — 3. Unfavorable (didn't like it/irritated)
**D15**	**Maoni yako kuhusu idadi ya ujumbe (SMS) hizo ni yepi?**<br>What did you think about the frequency of the messages that you received?<br>☐ 1. Chache sana — 1. Too few<br>☐ 2. Sawa — 2. Just right<br>☐ 3. Nyingi sana — 3. Too many
**D16**	**Je ulimtumia mtu yeyote ujumbe (SMS) hizo kuhusu kampeni ya chanjo?**<br>Did you forward or share a text message (SMS) about the vaccination campaign?<br>☐ 1. Ndio — 1. Yes<br>☐ 2. La — 2. No
**D17**	**Je, kuna rafiki au familia aliyekueleza au kukutumia ujumbe mfupi (SMS) kuhusu kampeni ya chanjo?**<br>Did a friend or family member forward or tell you about a text message (SMS) about the vaccination campaign?<br>☐ 1. Ndio — 1. Yes<br>☐ 2. La — 2. No

D18	**Je, ujumbe mfupi (SMS) ilikusaidia katika uamuzi wa kupata chanjo kwa mtoto wako?**<br>Was a text message (SMS) useful in making your decision to vaccinate your child?<br>☐ 1. Ndio — 1. Yes<br>☐ 2. La *[enda E1]* — 2. No *[skip to E1]*
**D19**	**Ujumbe (SMS) huo ulikusaidia vipi katika kufanya uamuzi kuhusu chanjo ya mtoto wako? *[Chagua zote zinazotajwa]***<br>How did text messages (SMS) help you make a decision to vaccinate your child? (Select all that apply.)<br>☐ 1. Ilinifahamisha kuhusu kampeni — 1. Made aware of the campaign<br>☐ 2. Ilinielezea wapi/wakati chanjo itapeanwa — 2. Provided place/ time for campaign vaccination<br>☐ 3. Ilinikumbusha kupeleka watoto kwenye chanjo — 3. Reminded to bring children for campaign vaccination<br>☐ 4. Iliondoa hofu kuhusu chanjo — 4. Relieved concerns about vaccination<br>☐ 5. Ilinieleza umuhimu wa chanjo — 5. Explained importance of receiving vaccine<br>☐ 6. Ilihakikisha chanjo inapeanwa na shirika inayoaminika (Wizara ya Afya) — 6. Verified that vaccine is provided by trusted source (MOH)<br>☐ 7. Ingine, eleza: _________________ — 7. Other (specify) __________________
**Sehemu ya E: Kuhusu familia** / Section E: Household characteristics	
**Kwa kumalizia, ningependa kukuuliza kuhusu familia yako**<br>"Finally, I'd like to ask some information about the household."	
E1	**Kiwango cha juu cha masomo ya mama/Mlezi ni kipi?**<br>What is the highest level of education attained by the mother/caretaker?<br>☐ 1. Hajasoma — 1. No education<br>☐ 2. Shule ya msingi, hajakamilisha — 2. Primary education- not completed<br>☐ 3. Shule ya msinigi kamilifu — 3. Primary education- completed<br>☐ 4. Shule ya upili — 4. Secondary<br>☐ 5. Zaidi ya shule ya upili — 5. Post-secondary<br>☐ 6. Madrasa — 6. Madrasa<br>☐ 7. Ingine, eleza:____________________ — 7. Other (specify) ________________
E2	**Je, unaweza kuzungumza na kuandika lugha ngapi? *[Chagua zote zinazotajwa]***<br>What languages can the mother/caretaker read and write? *[Select all that apply]*<br>☐ 1. Hakuna — 1. None<br>☐ 2. Kiswahili — 2. Kiswahili<br>☐ 3. Kingeresa — 3. English<br>☐ 4. Kiborana — 4. Borana<br>☐ 5. Kiembu — 5. Embu<br>☐ 6. Kikalenjin — 6. Kalenjin<br>☐ 7. Kikamba — 7. Kamba<br>☐ 8. Kikikuyu — 8. Kikuyu<br>☐ 9. Kikisii — 9. Kisii<br>☐ 10. Kiluhya — 10. Luhya<br>☐ 11. Kidhuluo — 11. Luo<br>☐ 12. Kimaragoli — 12. Maragoli<br>☐ 13. Kimaasi — 13. Maasai<br>☐ 14. Kimeru — 14. Meru<br>☐ 15. Kimijikenda — 15. Mijikenda<br>☐ 16. Kipokot — 16. Pokot<br>☐ 17. Kisomali — 17. Somali<br>☐ 18. Kiturkana — 18. Turkana<br>☐ 19. Ingine (eleza) _______________________ — 19. Other (ingine) ____________________

**E3**	**Mkuu wa nyumba anfanya kazi gani? (Chagua moja)**<br>What is the occupation of the head of the household? *[Choose one]*<br>☐ 1. Mkulima<br>☐ 2. Mchungaji mifugo<br>☐ 3. Ajira binafsi<br>☐ 4. Ajira<br>☐ 5. Hana kazi<br>☐ 6. Amestaafu<br>☐ 7. Mwanafunzi<br>☐ 8. Ingine (eleza): ________________________	 <br>1. Subsistence farming<br>2. Pastoralist<br>3. Self-employed<br>4. Formal employment<br>5. Unemployed<br>6. Retired<br>7. Student<br>8. Other (specify) ____________________
**E4**	**Kiwango cha juu cha masomo cha Mkuu wa nyumba ni kipi?**<br>What is the highest level of education attained by the head of the household?<br>☐ 1. Hajasoma<br>☐ 2. Shule ya msingi, hajakamilisha<br>☐ 3. Shule ya msinigi kamilifu<br>☐ 4. Shule ya upili<br>☐ 5. Zaidi ya shule ya upili<br>☐ 6. Madrasa<br>☐ 7. Ingine, eleza:____________________	 <br>1. No education<br>2. Primary education- not completed<br>3. Primary education- completed<br>4. Secondary<br>5. Post-secondary<br>6. Madrasa<br>7. Other (specify) ________________
**E5**	**Je, mkuu wa nyumba ni wa dini gani? *[Chagua moja]***<br>What is the religion of the household head? *[Choose one]*<br>☐ 1. MKatoliki<br>☐ 2. Mkristo (protestant)<br>☐ 3. Wakristo wengine<br>☐ 4. Mwislamu<br>☐ 5. Mhindu<br>☐ 6. Hakuna<br>☐ 7. Ingine, eleza:______________________	 <br>1. Catholic<br>2. Protestant<br>3. Other Christian<br>4. Muslim<br>5. Hindu<br>6. None<br>7. Other (specify):______________________
**E6**	**Je nyumba hii ina vyumba vingapi vya kulala**<br>How many separate rooms for sleeping are in the main dwelling unit?<br><br>**Idadi** / Number _________	
**E7**	**Je, sakafu ya nyumba imetengenezwa kwa kutumia nini? *[Chagua moja]***<br>What is the MAIN type of flooring for your house? *[Choose one.]*<br>☐ 1. Simiti<br>☐ 2. Udongo<br>☐ 3. Chaa cha ng'ombe<br>☐ 4. Tiles<br>☐ 5. Mbao<br>☐ 6. Mbao (imepakwa vanish/polish)<br>☐ 7. PCV<br>☐ 8. Carpet<br>☐ 9. Ingine (eleza): ________________________	 <br>1. Cement<br>2 Earth/sand<br>3. Mud/cowdung<br>4. Ceramic tiles<br>5. Unfinished wood/planks<br>6. Polished wood<br>7. Vinyl/asphalt strips<br>8. Carpet<br>9. Other (specify) ________

**E8**	**Je, kuta za nyumba hii zimetengenezwa kwa kutumia nini?** ***[Chagua moja]***<br>**What is the main type of materials used for the walls for your house? [Choose one]**<br>☐ 1. Simiti — 1. Cement/Bricks<br>☐ 2. Udongo/samadi — 2 Mud/cowdung<br>☐ 3. Mawe — 3. Stone<br>☐ 4. Nyasi/Makuti — 4. Grass/makuti<br>☐ 5. Mbao — 5. Wood<br>☐ 6. Mabati — 6. Iron sheets<br>☐ 7. Tiles — 7. Tiles<br>☐ 8. Ingine,(eleza) — 8. Other (specify) ________
**E9**	**Je, paa ya nyumba hii imetengenezwa kwa kutumia nini?** ***[Chagua moja]***<br>**What is the main type of material used for the roof for your house? [Choose one]**<br>☐ 1. Simiti — 1. Cement/Bricks<br>☐ 2. Udongo/Samadi — 2 Mud/cowdung<br>☐ 3. Mawe — 3. Stone<br>☐ 4. Nyasi/Makuti — 4. Grass/makuti<br>☐ 5. Mbao — 5. Wood<br>☐ 6. Mabati — 6. Iron sheets<br>☐ 7. Tiles — 7. Tiles<br>☐ 8. Ingine (Eleza) — 8. Other (specify) ________
**E10**	**Je mnapata mwangaza kwa kutumia nini?** ***[Chagua moja]***<br>What is your MAIN source of lighting? *[Choose one.]*<br>☐ 1. Stima — 1. Electricity<br>☐ 2. Nishati ya jua — 2. Solar<br>☐ 3. Kuni — 3. Firewood<br>☐ 4. Mafuta taa — 4. Kerosene<br>☐ 5. Gesi — 5. Gas<br>☐ 6. Mshumaa — 6. Candles<br>☐ 7. Ingine (eleza) _____________ — 7. Other (specify) _____________
**E11**	**Mnatumia nini kupika?** ***[Chagua moja]***<br>What is your MAIN source of cooking fuel? *[Choose one.]*<br>☐ 1. Kuni — 1. Firewood<br>☐ 2. Makaa — 2. Charcoal<br>☐ 3. Mafuta taa — 3. Kerosene<br>☐ 4. Stima — 4. Electricity<br>☐ 5. Gesi — 5. Gas<br>☐ 6. Ingine (eleza):___________________ — 6. Other (specify): ____________________
**E12**	**Je, mko na shamba ya kupanda vyakula?**<br>Does the household own agricultural land?<br>☐ 1. Ndio — 1. Yes<br>☐ 2. La — 2. No
**E13**	**Je, mnazo vifaa gani kati ya hivi?** ***[Chagua zote zinazotajwa]***<br>Which of the following items does the household own? *[Check all that apply]*<br>☐ 1. Saa — 1. Clock/watch<br>☐ 2. Redio — 2. Radio<br>☐ 3. Televisheni — 3. TV<br>☐ 4. Meza — 4. Table<br>☐ 5.Sofa — 5. Sofa<br>☐ 6. Kabati — 6. Cupboard<br>☐ 7. Baisikeili — 7. Bicycle<br>☐ 8. Pikipiki — 8. Motorcycle/scooter<br>☐ 9. Gari/lori — 9. Car/truck

Household serial number ___ ___ ___ ___–___ ___ ___

**E14**	**Je,umewahi kusikia kuhusu mnyoo wa mkanda**<br>Have you heard of guinea worm?<br>☐ 1. Ndio 1. Yes<br>☐ 2. La 2. No
**E15**	**Je, umewahi kusikia kuhusu zawadi ya Shilingi laki moja kwa watu wanaoripoti matukio ya mnyoo wa mkanda?**<br>Have you heard of the 100,000 KSH cash reward for reporting suspected cases of Guinea Worm?<br>☐ 1. Ndio 1. Yes<br>☐ 2. La 2. No

Asante kwa kushiriki kwa mahojiano hii. Je, unayo maswali yoyote?
[Jibu maswali yote ya mhojiwa]

Thank you for participating in this survey. Do you have any questions for me?
[Answer all questions participant may have]
