# Supplementary material for: Use of the revised World Health Organization cluster survey methodology to classify measles-rubella vaccination campaign coverage in 47 counties in Kenya, 2016
Source: PLoS One. 2018 Jul 2;13(7):e0199786. doi: 10.1371/journal.pone.0199786 (PMC6028100; doi:10.1371/journal.pone.0199786)
Supplement: S1 Fig — (PDF) [file pone.0199786.s004.pdf]

Measles-rubella post-vaccination campaign coverage survey in Kenya, 2016

Fig S1. Organ-pipe plots of unweighted measles-rubella campaign vaccination coverage by cluster and county — Kenya 2016 (14). For each cluster, the total number of eligible children aged 9 months–14 years that were enrolled in the survey is printed at the bottom of the bar. Out of the total 720 clusters selected for the survey, 5 clusters were inaccessible (I), and 2 clusters had no eligible (NE) children for inclusion; these clusters are indicated with I or NE printed at the bottom of the bar. Overall, 349 clusters had 100% coverage; 156 had 95–99% coverage; 119 had 90–94% coverage; 41 had 85–89% coverage; 23 had 80–84% coverage; 6 had 75–79% coverage; 6 had 70–74% coverage; 11 had <70% coverage. One cluster each in Nairobi and Wajir had 0% coverage. In the Nairobi cluster with 0%, there was only one eligible child, who was unvaccinated. In the Wajir cluster with 0%, there were three eligible households with a total of 12 unvaccinated children. Reasons for non-vaccination in these two clusters included being unaware of the campaign (9/13) and being unaware of the need for vaccination (1/13).

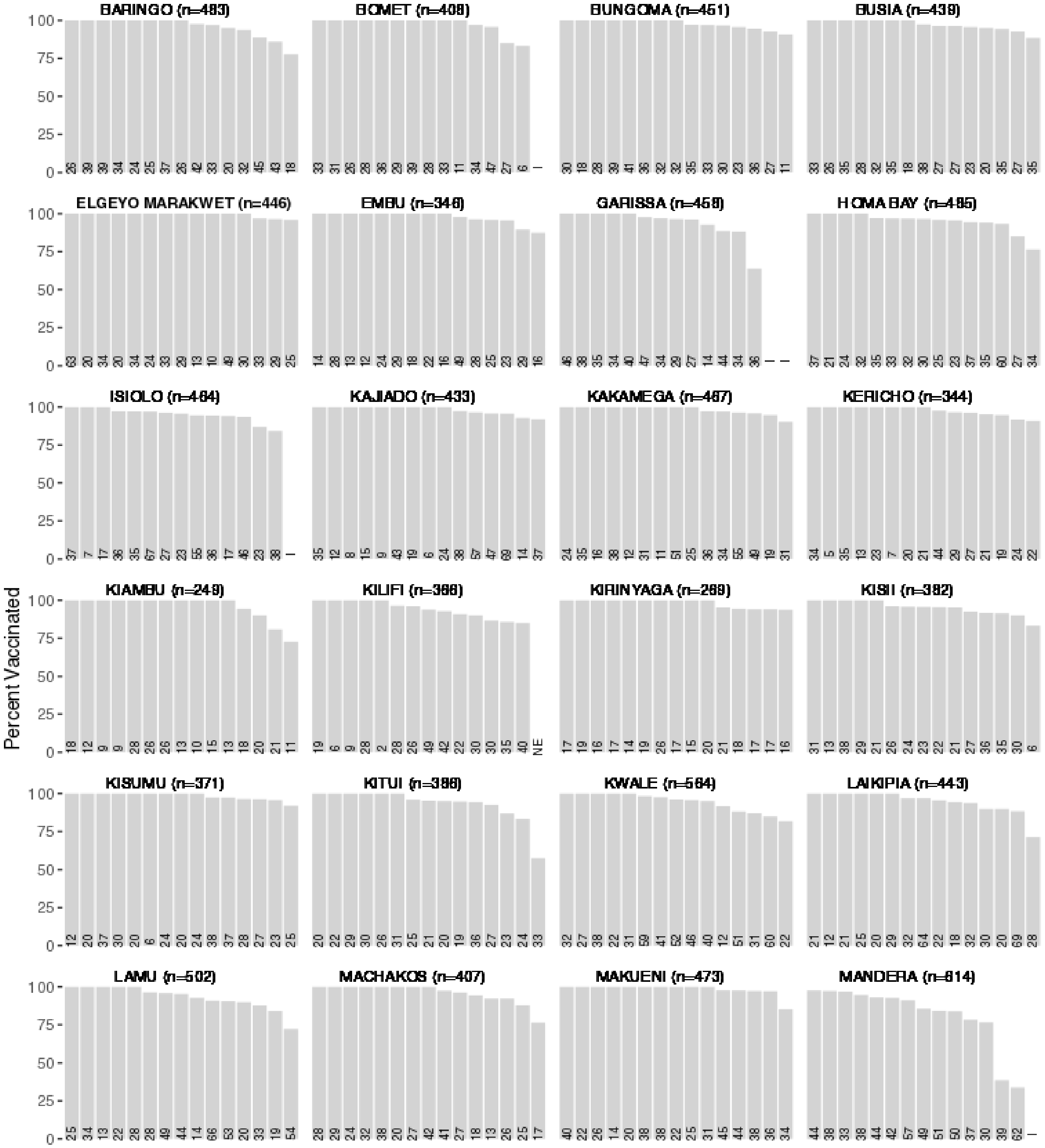

Percent Vaccinated
BARINGO (n=483)
BOMET (n=408)
BUNGOMA (n=451)
BUSIA (n=439)
ELGEYO MARAKWET (n=446)
EMBU (n=348)
GARISSA (n=458)
HOMA BAY (n=485)
ISIOLO (n=464)
KAJIADO (n=433)
KAKAMEGA (n=467)
KERICHO (n=344)
KIAMBU (n=249)
KILIFI (n=366)
KIRINYAGA (n=269)
KISII (n=382)
KISUMU (n=371)
KITUI (n=386)
KWALE (n=564)
LAIKIPIA (n=443)
LAMU (n=502)
MACHAKOS (n=407)
MAKUENI (n=473)
MANDERA (n=614)
100
75
50
25
0

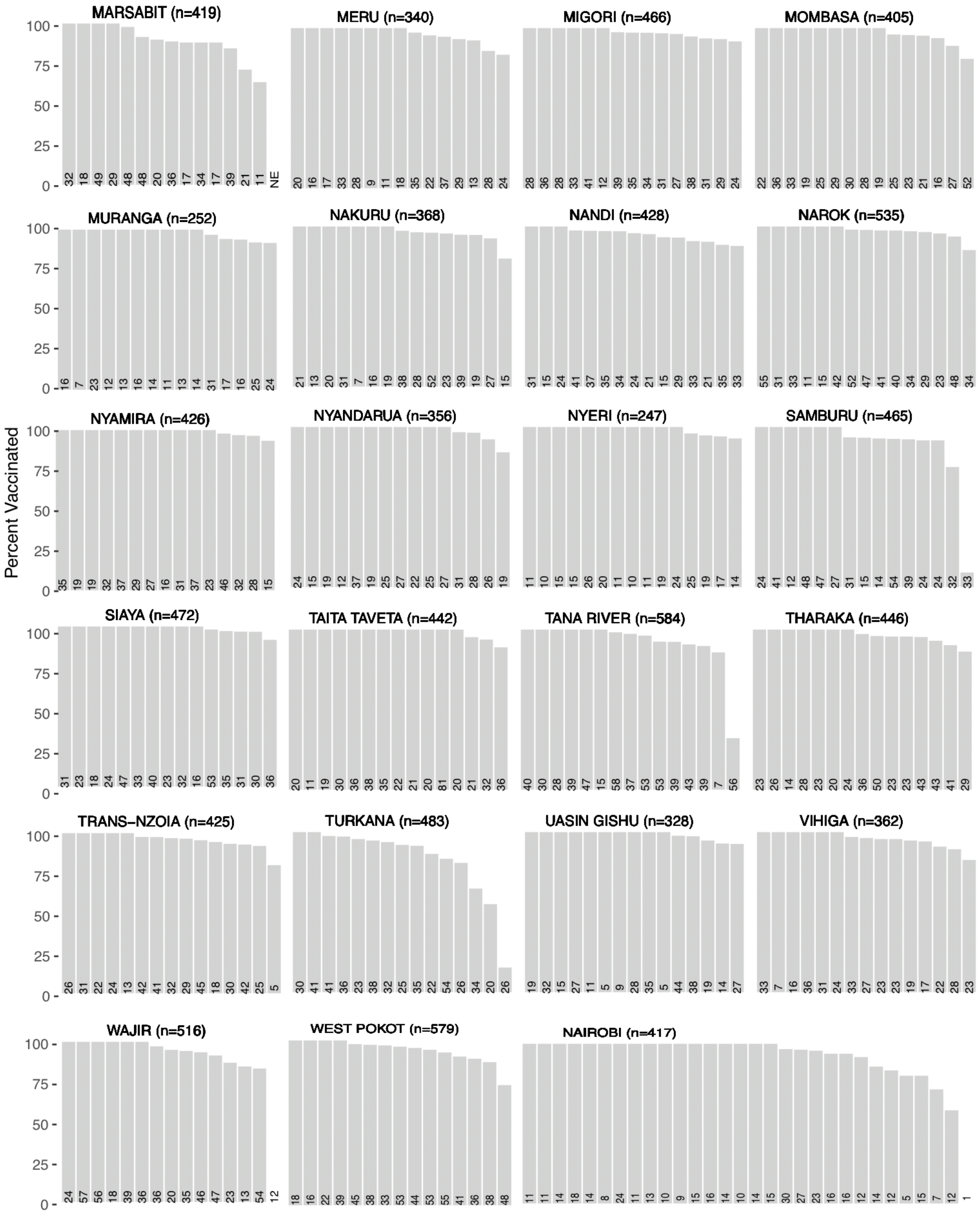
Percent Vaccinated
MARSABIT (n=419)
MERU (n=340)
MIGORI (n=466)
MOMBASA (n=405)
MURANGA (n=252)
NAKURU (n=368)
NANDI (n=428)
NAROK (n=535)
NYAMIRA (n=426)
NYANDARUA (n=356)
NYERI (n=247)
SAMBURU (n=465)
SIAYA (n=472)
TAITA TAVETA (n=442)
TANA RIVER (n=584)
THARAKA (n=446)
TRANS-NZOIA (n=425)
TURKANA (n=483)
UASIN GISHU (n=328)
VIHIGA (n=362)
WAJIR (n=516)
WEST POKOT (n=579)
NAIROBI (n=417)
